# Proceedings of the 9th international symposium on veterinary rehabilitation and physical therapy

**DOI:** 10.1186/s13028-016-0259-7

**Published:** 2016-12-14

**Authors:** Elodie Nemery, Annick Gabriel, Dominique Cassart, Calixte Bayrou, Joëlle Piret, Nadine Antoine, Monika Nilsson, Lars Steinwall, Inger Jacobson, Ângela Martins, Carla Carvalho, Inês Viegas, Denis J. Marcellin-Little, Ola L. A. Harrysson, Christopher S. Crimi, David Levine, María Calatayud, María Resano, Marion Mucha, Ivonne Virac, Cornelia Lang, Kathleen Wittek, Alexander Tichy, Barbara Bockstahler, J. Randy Walker, Āren Swogger, Tavis Gibson, Janice Ryan, Chris Gilligan, Katie Haulcomb, Leigh Anne Norris, Matt Powers, Tracy Pugh, Seth Purkey, Hanna Pulkkinen, Anu Lappalainen, Outi Laitinen-Vapaavuori, Heli Hyytiäinen, Ann Essner, Rita Sjöström, Lena Zetterberg, Karin Hellström, Pia Gustås, Hans Högberg, Anna Hielm-Björkman, Charlotte Orrfors, Gunnevi Sundelin, Luísa Gonçalves, João Niza-Ribeiro, Darryl L. Millis, Augusto José de Matos, Marinette Teeling, Kate Ross, Victoria Geddes, Ann Carstens, Tineka Kriel, Karien du Toit, Jeanette Pauw, Gillian Martindale, Kristine Mylo, Sybrand S. van den Berg, Morito Ogasawara, Hiromi Noguchi, Takeo Minami, Krzysztof Zdeb, Urszula Baumgart, Ana M. Ribeiro, Ricardo Palas, Martinho Capelão, Mila Speciani, Alessandra De Luca, Elisa Anzolin, Nina Pirinen, Matti Pastell, Anna Mykkänen, Jonna Jokisalo, Kati Niinistö, Laura Hänninen, Catherine McGowan, Alexandria Holt, Marta Subirats, Maria Perez, Tatiana Hernández, Luna Gutierrez-Cepeda, Rafael Cediel, Javier López-San Román, Anna F. Boström, Lotta Savolainen, Anu K. Lappalainen, Sarah Stadig, Linda Lundström, Anna Bergh, Charles Ley, Lena Olsén, Carina Ingvast-Larsson, Renata Diniz, Cristina Nicolau, Antonio Gamundi, Mourad Akaarir, Elizabeth Roberts, Leander McLennan, Helen C. Cartildge, Lucy K. M. Evans, Stephen Baugh, Pernilla Stenfeldt, Cajsa Ericson, Linnéa Söderberg, Lennart Sjöström, Robert Colborne, Anna Byström, Marti Drum, Marie de Swarte, Federica Morandi, José Guevara, Dawn Hickey, Ellen Camp, Rachel Dickson

**Affiliations:** 1 Faculty of Veterinary Medicine, FARAH Research Center, University of Liège, Sart Tilman, Liège, Belgium; 2 Division of Health Sciences, Luleå University of Technology, 971 87 Luleå, Sweden; 3 Department of Veterinary Sciences, University of Lusófona de Humanidades e Tecnologias, Campo Grande 376, 1749-024 Lisboa, Portugal; 4 North Carolina State University, Raleigh, NC USA; 5 Department of Physical Therapy, The University of Tennessee at Chattanooga, Chattanooga, TN USA; 6 HorseRehab, 46111 Valencia, Spain; 7 Department for Small Animals and Horses, Small Animal Surgery, Section for Physical Therapy and Rehabilitation, University of Veterinary Medicine, 1210 Vienna, Austria; 8 Department for Biomedical Sciences, Platform Bioinformatics and Biostatistics, University of Veterinary Medicine, 1210 Vienna, Austria; 9 Physical Therapy, Life Care Centers of America, Dayton, TN USA; 10 Star Physical Therapy, Clarksville, TN USA; 11 Siskin Hospital for Physical Rehabilitation, Chattanooga, TN USA; 12 Department of Equine and Small Animal Medicine, University of Helsinki, Helsinki, Finland; 13 Department of Neuroscience, Uppsala University, Uppsala, Sweden; 14 Evidensia Djurkliniken Gefle, Gävle, Sweden; 15 Region Jämtland Härjedalen, Unit of Research Education and Development, Östersund, Sweden; 16 Department of Community Medicine and Rehabilitation Physiotherapy, Umeå University, Umeå, Sweden; 17 Department of Clinical Sciences, Swedish University of Agricultural Sciences, Uppsala, Sweden; 18 Department of Health and Caring Sciences, University of Gävle, Gävle, Sweden; 19 Fysiohund Sverige, Stockholm, Sweden; 20 Abel Salazar Institute for the Biomedical Sciences-ICBAS, University of Porto, Porto, Portugal; 21 Department of Population Studies, ICBAS and Institute of Public Health (ISPUP), University of Porto, Porto, Portugal; 22 Department of Small Animal Clinical Sciences, University of Tennessee College of Veterinary Medicine, Knoxville, Tennessee USA; 23 Animal Science and Study Centre/Food and Agrarian Sciences and Technologies Institute (CECA/ICETA), University of Porto, Porto, Portugal; 24 Equine-Librium College, Plettenberg Bay, Western Cape, 6600 South Africa; 25 University of Pretoria, Pretoria, 0110 South Africa; 26 Bayside Animal Clinic, Yokohama, Kanagawa Japan; 27 Shijonawate Gakuen University, Daito, Osaka Japan; 28 Minami Animal Hospital, Igaueno, Mie Japan; 29 Legwet Veterinary Clinic, Legionowo, Masovia Poland; 30 Physical Therapy Service, Pet Restelo Fisio & Spa Restelo, Restelo, 1400-195 Lisboa, Portugal; 31 Neurology Service, Hospital Veterinário do Restelo, Restelo, 1400-195 Lisboa, Portugal; 32 Pet Restelo Fisio & Spa Restelo, 1400-195 Lisboa, Portugal; 33 Freelance Veterinarian, Mantua, Italy; 34 KinesioProject asd, Bari, Italy; 35 Ambulatorio Veterinario Anzolin, Verona, Italy; 36 Department of Equine and Small Animal Medicine, Faculty of Veterinary Medicine, University of Helsinki, Helsinki, Finland; 37 Natural Resources Institute of Finland (Luke), Green Technology, Helsinki, Finland; 38 Evidencia Hyvinkään Hevossairaala, Hyvinkää, Finland; 39 Department of Production Animal Medicine, Faculty of Veterinary Medicine, University of Helsinki, Helsinki, Finland; 40 Department of Musculoskeletal Biology, Institute of ageing and chronic disease, University of Liverpool, Liverpool, UK; 41 TN Department of Clinical Sciences, North Carolina State University, Raleigh, NC 27607 USA; 42 Small Animals Physiotherapy Service, Firvet Fisioterapia y Rehabilitación VeterinariaPolinyà, 08213 Barcelona, Spain; 43 Department of Clinical Science Neurology/Neurosurgery Service, College of Veterinary Medicine, Mississippi State University, Starkville, MS 39760 USA; 44 Fisioveterinaria, 28523 Madrid, Spain; 45 Universidad Complutense de Madrid, 28040 Madrid, Spain; 46 Small Animal Surgery, Department of Equine and Small Animal Medicine, Faculty of Veterinary Medicine, University of Helsinki, Helsinki, Finland; 47 Hälsinge Small Animal Clinic, Hudiksvall, Sweden; 48 Department of Anatomy, Physiology and Biochemistry, Swedish University of Agricultural Sciences, Uppsala, Sweden; 49 Division of Pharmacology and Toxicology, Department of Biomedical Sciences and Veterinary Public Health, Swedish University of Agricultural Sciences, Uppsala, Sweden; 50 Department of Biology, University of the Balearic Islands, Mallorca, Spain; 51 Animal Production, Welfare and Veterinary Sciences Department, Harper Adams University, Edgmond, Shropshire TF10 8NB UK; 52 Veterinary Physiotherapy, University of Liverpool, Leahurst, Liverpool, CH64 7TE UK; 53 Hästrehab & Byggkonsult I Ängelholm AB, Vanstadsvägen 121, 262 91 Ängelholm, Sweden; 54 Animotion Rehab, Ankdammsgatan 18, 171 43 Solna, Sweden; 55 Department of Clinical Sciences, Faculty of Veterinary Medicine and Animal Husbandry, Swedish University of Agricultural Sciences, Box 7054, 750 07 Uppsala, Sweden; 56 Evidensia Strömsholm Referral Animal Hospital, Djursjukhusvägen 11, 734 94 Strömsholm, Sweden; 57 Institute of Veterinary, Animal and Biomedical Sciences, Massey University, Private Bag 11-222, Palmerston North, 4442 New Zealand; 58 Department of Anatomy, Physiology and Biochemistry, Faculty of Veterinary Medicine and Animal Husbandry, Swedish University of Agricultural Sciences, Box 7011, 750 07 Uppsala, Sweden; 59 Department of Small Animal Clinical Sciences, Canine Arthritis, Rehabilitation, Exercise and Sports Medicine (CARES) Section, University of Tennessee College of Veterinary Medicine, Knoxville, TN USA; 60 Department of Small Animal Clinical Sciences, Radiology Section, University of Tennessee, Knoxville, TN USA

## A1 Equine menisci in osteoarthritis: preliminary results in histological examination

### Elodie Nemery, Annick Gabriel, Dominique Cassart, Calixte Bayrou, Joëlle Piret, Nadine Antoine

#### Faculty of Veterinary Medicine, FARAH Research Center, University of Liège, Sart Tilman, Liège, Belgium

##### **Correspondence:** Elodie Nemery - elodie.nemery@ulg.ac.be

*Acta Veterinaria Scandinavica* 2016, **58(Suppl 2)**:A1

**Background:** In equine species, meniscal tears are frequent as soft tissue injuries in the stifle and can be responsible for the premature ending of careers of sporting horses due notably to the concomitant presence of degenerative articular disease [1, 2]. In humans, Pauli and collaborators [3] demonstrated a correlation between osteoarthritis grades and meniscal degeneration.

**Objectives:** Our objective was to evaluate the putative macroscopical and histological changes on equine menisci with various grades of stifle osteoarthritis.

**Materials and methods:** Menisci were taken at necropsy, 1–48 h post mortem, from two mares aged 11 and 13 years, respectively and with body weight of approximately 590 kg. Macroscopical and microscopical grading systems were used (Table 1) to establish (i) macroscopical chondropathy scores, (ii) macroscopical degenerative meniscal state and to evaluate, using specific colorations, (iii) the collagen network organization and (iv) the content of proteoglycans in the matrix of the menisci. Proteoglycans were revealed with Safranin-O, the collagen fibers organization with Fast Green and cell’s nuclei with Weigert’s Iron *Hematoxylin*.

**Table 1:** Macroscopical and microscopical grading system

	Macroscopic assessment		Histologic assessment	
	Chondropathy score	Menisci examination	Collagen networks	Content of proteoglycans
Based on	[4]	[3]	[5]	[5]
Grading system	Grade 0 = normal → Grade 4 = severe chondropathy	Grade 1 = normal menisci → Grade 4 = degenerated menisci	Normal = homogen green, organized matrix of collagen → Pathologic = disrupted, less organized, green matrix of collagen	Grade 0 = minimal red staining → Grade 5 = strong red staining

**Fig. 1:**
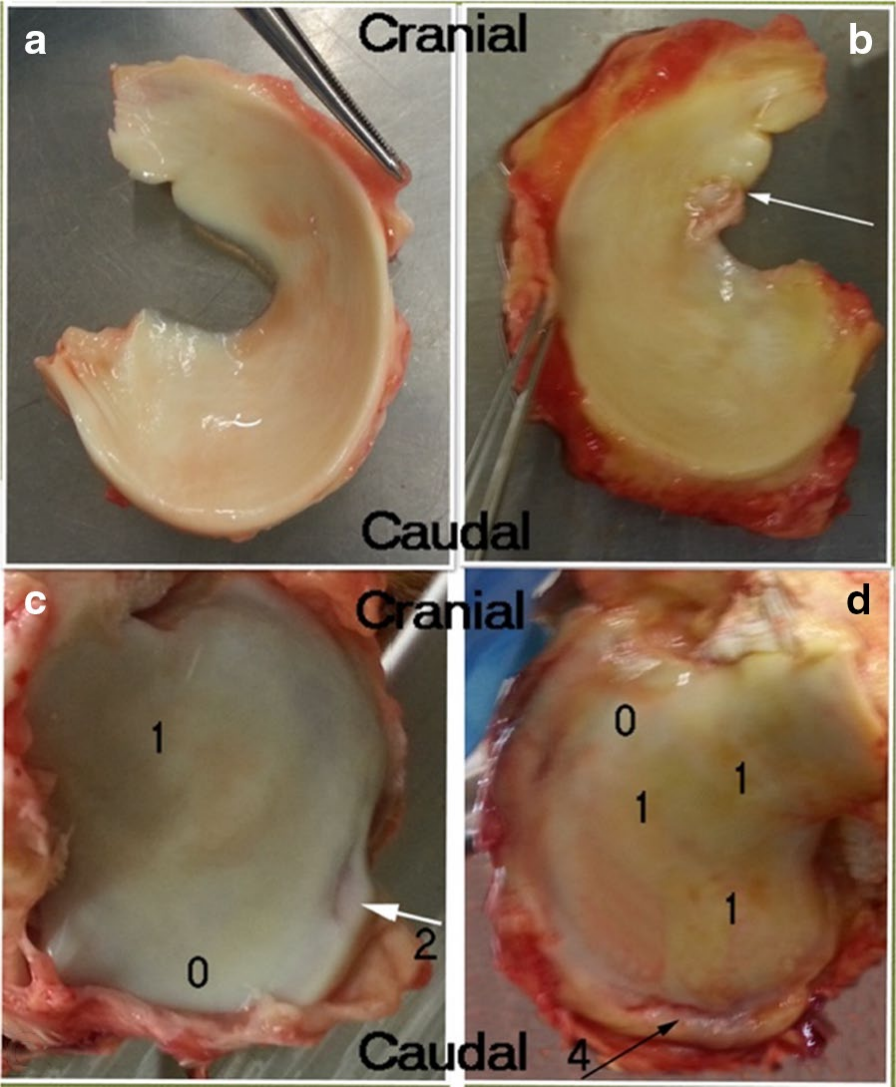
Macroscopic examination of the medial menisci and of the articular cartilage surface on the medial tibial plateau of a “low” (**a–c**) and a “high chondropathy horse” (**b–d**). **c**, **d** 0–4: Chondropathy scores established following Ashraf and collaborators [4]

**Results:** Macroscopically, the “low chondropathy horse” (LCH) (Fig. 1a–c) revealed in its medial tibial plateau a grade 2 “lightly broken surface, white to off-white in colour” (arrow, Fig. 1c) whereas the “high chondropathy horse” (HCH) (Fig. 1b–d) exhibited a grade 4 “subchondral bone exposure, red in colour” (arrow, Fig. 1d). For both, a “swelling and softening of the cartilage with a light brown homogeneous coloration” (grade 1) was observed with a more extended region in “HCH” than in “LCH”.The normal cartilage (grade 0) was much reduced in the “HCH”. The “LCH” (Fig. 1a) had a normal grade 1 meniscus and the “HCH” a grade 2 exhibiting a frayed inner border (arrow, Fig. 1b).

**Fig. 2:**
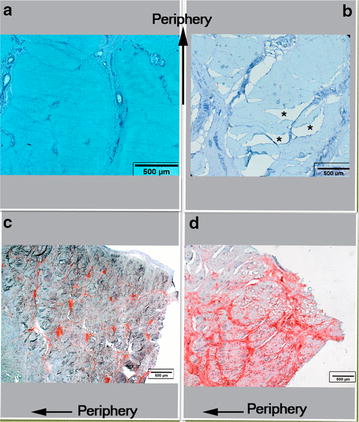
Classical coloration with Safranin-O, fast green and Weigert’s iron hematoxylin of medial menisci belonging to a “low” (**a–c**) and a “high chondropathy horse” (**b–d**). *Cleft in the collagen matrix; the collagen networks of the menisci was abnormally disrupted in the “HCH”

On histological sections (Fig. 2), the collagen networks of the menisci was abnormally disrupted in the “HCH” (Fig. 2b) compared to the LCH (Fig. 2a). A grade 3 was attributed to the LCH (Fig. 2c) regarding the intensity of Safranin O proteoglycans coloration whereas the “HCH” exhibited a grade 5 (Fig. 2d).

**Conclusions:** In conclusion, meniscal degeneration was correlated with the grade of chondropathy and although these results are preliminary due to the small sample size, the finding is nevertheless interesting as, to the authors’ knowledge, our work describes for the first time the microscopical change of the equine menisci related to a low and a high chondropathy score.

**Trial registration:** Not applicable. This is not a research study that “prospectively assigns human participants or groups of humans to one or more health-related interventions to evaluate the effects on health outcomes.”

**Consent to publish:** Not applicable. This is not a study performed on humans.

**References**

1. Walmsley JPR, Phillips TJ, Townsend HGG. Meniscal tears in horses: an evaluation of clinical signs and arthroscopic treatment of 80 cases. Equine Vet J. 2003; 35:402–6.

2. De Busscher V, Verwilghen D, Bolen G, Serteyn D, Busoni V. Meniscal damage diagnosed by ultrasonography in horses: a retrospective study of 74 femorotibial joint ultrasonographic examinations (2000–2005). J Equine Vet Sci. 2006; 26:453–61.

3. Pauli C, Grogan SP, Patil S, Otsuki S, Hasegawa A, Koziol J, et al. Macroscopic and histopathologic analysis of human knee menisci in aging and osteoarthritis. Osteoarthritis Cartilage. 2011; 19:1132–41.

4. Ashraf S, Wibberley H, Mapp PI, Hill R, Wilson D, Walsh DA. Increased vascular penetration and nerve growth in the meniscus: a potential source of pain in osteoarthritis. Ann Rheum Dis. 2011; 70:523–9.

5. Sun Y. Histological examination of collagen and proteoglycan changes in osteoarthritic menisci. Open Rheumatol J. 2012; 6:24–32.

## A2 Effect of acupuncture treatment for a canine with hip osteoarthritis—a study in single subject experimental design

### Monika Nilsson, Lars Steinwall, Inger Jacobson

#### Division of Health Sciences, Luleå University of Technology, Sweden

##### **Correspondence:** Inger Jacobson - inger.jacobson@ltu.se

*Acta Veterinaria Scandinavica* 2016, **58(Suppl 2)**:A2

**Background:** Hip osteoarthritis in canines is a common diagnosis. The prevalence in adult dogs is estimated to be 20 percent. The primary treatment is usually an NSAID. Acupuncture as treatment for pain conditions is commonly used within human medicine and is becoming more frequently used within veterinary medicine. Acupuncture studies that show the pain relieving effects in animals are few, which make it important to elucidate the effects of this treatment method.

**Objectives:** The aim of this study was to examine the effects of acupuncture in relation to function, passive range of motion, thigh circumference and palpation for pain in a ten year-old German Shepard dog with x-ray verified left sided osteoarthritis of the hip.

**Materials and methods:** The study was conducted with a single subject experimental ABA-design. Twice before the first treatment (A1-baseline) function, passive range of motion (PROM), thigh circumference and palpation for pain were assessed. The dog then received three acupuncture treatments, once a week (B1–3-intervention). The choice of points in order of insertion was Bai Hui intraspinal L7–S1, BL 25 bilateral, BL 23 bilateral, and GB 30 left. The needles were stimulated during insertion as well as before removal. During intervention, PROM was measured after each treatment. Seven days after the last acupuncture treatment the same examination that was conducted initially was conducted again (A2-evaluation). Data were analyzed with 2SD-line where all the results that orient above 2SD-line are significant. Celeration line shows the trend before, during and after the intervention.

**Fig. 3:**
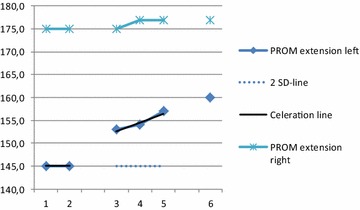
Passive hip extension in degrees. Baseline examination (1, 2), during intervention (3–5) and after the intervention (6). The trend is clear with an improved range of motion in the primary outcome measure and 2SD-line show a significant result

**Results:** The results showed decreased symptoms regarding limpness and rigidity and normalized functional tests, improved passive range of motion in the left hip joint (Fig. 3), decreased pain of the outer part of passive extension, increased thigh circumference bilaterally, and decreased pain with palpation.

**Conclusions:** The single subject experimental design has great clinical applicability for the scientist-practitioner and can provide clinicians with useful information. This single subject experimental design indicates that acupuncture seems to have an effect on dogs with hip osteoarthritis. Further studies with larger study groups need to be conducted to be able to make evidence-based treatment decisions.

## A3 Functional neurorehabilitation in dogs with peripheral vestibular syndrome

### Ângela Martins, Carla Carvalho, Inês Viegas

#### Department of Veterinary Sciences, University of Lusófona de Humanidades e Tecnologias, Campo Grande 376, 1749-024, Lisboa, Portugal

*Acta Veterinaria Scandinavica* 2016, **58(Suppl 2)**:A3

**Background:** Functional neurorehabilitation protocol (FNRP) are not implemented nor reported in most dogs with peripheral vestibular syndrome (PVS). The FNRP applied was based on the neuroanatomy/physiology of PVS.

**Objectives**: Determine if benefits exist with implementing a FNRP into treatment for dogs with PVS; if their progress is remarkable when compared to those whose treatment plan did not include a FNRP; and compare the difference between a protocol with a treatment plan that did not include a FNRP, but includes a treatment with corticosteroids, and also to verify if age, weight and etiology influence final results.

**Materials and methods:** Dogs (n = 21) diagnosed with PVS at any age, gender, weight, etiology and treatment hospitalized for more than 5 days were included in this study after a neurologic and FNR evaluation. Every dog that showed a major improvement during the first 5 days were automatically removed from this study. The remaining stayed hospitalized during 21 days.

Three groups of study included:

- Group 1 FNRP and basic treatment (BT)
- Group 2 Administration of corticosteroids and BT
- Group 3 BT

The BT plan included fluid therapy, nutritional, water support and anti-emetic treatment. A pioneer scale, a balance scale, analogous to the human balance scale (Berg Balance Scale) was used to measure dogs weekly results. Statistical analysis (Microsoft Office Excel 2007, IBM SPSS Statistics 22.0 was used for analysis.

**Results:** The balance scale demonstrated all dogs included in the study improved over time (except for one that died). Group 1 yielded 96.4% improvement, followed by Group 3 (78.6%) Group 2 (65.4%). Differences between the three groups were observed from Day 14 with a 95% significance at day 21 between Group 1–2; Group 1–3 (F(2,18) = 5.084, P = 0.018). No statistical difference existed between Group 2 and 3. Age and etiology did not show any influence on the final outcome. Heavier animals had a slower recovery in the beginning, later evolving into the same recovery rates than lighter animals. Older dogs (Group 1) showed a better performance (P = 0.003).

**Conclusions:** Applying FNR in dogs with PVS has better results than the administration of corticosteroids or than only BT. The administration of corticosteroid does not have benefits in the final performance. Weight influenced the results. Age seems to influence the performance of animals that went through FNRP (older animals have better performance).

## A4 Influence of three tibial osteotomy procedures on the proximodistal patellar position in the cranial cruciate ligament-deficient stifle (knee) in the dog

### Denis J. Marcellin-Little^1^, Ola L. A. Harrysson^1^, Christopher S. Crimi^1^, David Levine^2^

#### ^1^North Carolina State University, Raleigh, NC, USA, ^2^Department of Physical Therapy, The University of Tennessee at Chattanooga, Chattanooga, TN, USA

##### **Correspondence:** David Levine - david-levine@utc.edu

*Acta Veterinaria Scandinavica* 2016, **58(Suppl 2)**:A4

**Background:** Cranial cruciate ligament (CCL) injuries are common in dogs and are often managed by tibial osteotomies. Little is known about the influence of osteotomies on the proximodistal patellar position (PDPP), which influences mechanics of the knee joint including the angle of the patellar ligament in the sagittal plane and constraint from trochlear ridges.

**Objectives:** To fabricate four accurate replicas of a canine pelvic limb, perform three corrective osteotomies of the tibia: tibial plateau leveling osteotomy (TPLO), tibial tuberosity advancement (TTA), and triple tibial osteotomy (TTO), and measure the influence of the osteotomies on the PDPP.

**Materials and methods:** CT scan and radiographs of the left pelvic limb of a Doberman Pinscher were used. 23 geometric markers were added to the tibia, femur, and patella to enhance the reproducibility of the physical models and assist in final assembly. The leg replicas were created using 3D printing, room temperature vulcanizing silicone molding, and casting. PDPP was measured in 5 joint positions (75°, 96°, 113°, 130°, and 148°) after removal of the CCL and after performing a TPLO, TTA, or TTO on leg replicas.

**Fig. 4:**
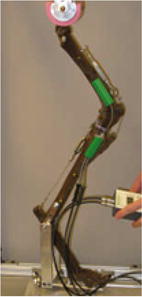
Articulated replica of a canine pelvic limb fabricated using 3D printing. The patellar ligament is made using braided silk. The leg is held up within a grame and the hip joint is loaded. Quadriceps femoris and gastrocnemius muscle tones are simulated using metal wires. The knee angle is assessed using an electrogoniometer

**Results:** Mean (±SD) bone length was 246 ± 0.5 mm for 5 femoral replicas, 254 ± 0.7 mm for 5 tibial replicas, and 29.4 ± 0.2 mm for 8 patellar replicas. The pes measured 237 mm. The tibial plateau slope was 28°. The CCL measured 27 mm, the CaCL and LCL 33 mm, the LCL 48 mm, and the patellar ligament 59 mm. The intact model maintained a constant stifle and hock joint angle when loaded without mediolateral or craniocaudal subluxation. The CCL-deficient model also maintained a constant stifle and hock joint angle when loaded with a cranial thrust of 0.6 mm at 75°, 7.2 mm at 96°, 17.1 mm at 113°, 8.1 mm at 130°, and 7.1 mm at 148°. CCL removal induced a mean distal patellar displacement of 2.6 ± 5.7% of patellar length. The TPLO induced a proximal patellar displacement of 6.6 ± 2.2%. The TTA induced a distal patellar displacement 12.7 ± 5.3%. The TTO induced a proximal patellar displacement of 10.0 ± 6.1% (Fig. 4).

**Conclusions:** The physical models accurately matched a canine pelvic limb, eliminated the variability present in cadaveric studies, and allowed the assessment of the effects of surgical procedures on the stability of the stifle joint and the motion of the patella. Tibial corrective osteomies lead to changes in patellar position: TTO and TPLO lead to proximal patellar displacement and TTA leads to distal patellar displacement.

## A5 Improvement of myofascial pain in equine brachiocephalicus muscle using dry needling technique, a clinical commentary

### María Calatayud, María Resano

#### HorseRehab, 46111, Valencia, Spain

##### **Correspondence:** María Calatayud - minuscula@movistar.es

*Acta Veterinaria Scandinavica* 2016, **58(Suppl 2)**:A5

**Background:** Myofascial pain has become a common diagnosis in human patients with myofascial trigger points (MTrPs). A MTrP is defined as a hyperirritable spot in a taut band of skeletal muscle fibers. Dry needling (DN) is a skilled intervention to treat MTrPs. One of the clinical findings associated with MTrPs includes a local twitch response (LTR), a spinal cord reflex characterized by an involuntary contraction of the taut band. DN diminishes persistent peripheral nociceptive input and improves function.

**Objectives:** The aim of this study is to investigate whether DN can be a technique to reduce myofascial pain due to MTrPs in equine brachiocephalicus muscle.

**Materials and methods:** Ten horses aged between 5 and 15 years old were examined. One was excluded due to dangerous behaviour. Manual palpation of the distal end of brachiocephalicus muscle was performed by the same operator. Two were excluded due to lack of a painful response. Pain threshold was measured using a pressure algometer (Wagner Instruments, USA, kg/cm^2^) 3 times: before treatment, and 1 and 72 h post treatment in the distal end of the muscle on both sides. At each testing point, the same operator applied the tip of the algometer parallel to the neck and transverse to the muscle fibers, consistently increasing the pressure until an escape movement or facial signs of pain were seen. DN was performed using a 30 × 40 mm Agupunt^®^ needle. The muscle was selected with a pincher palpation and needling was performed with a dynamic intramuscular stimulation, away from the Cave and caudal from C3–C4. LTR were elicited until eradicated and recorded. Repeated Measures ANOVA and T Test were used for statistical analysis.

**Results:** Seven horses demonstrated signs of discomfort when MTrPs were palpated in the distal end of both brachiocephalicus muscles. LTR were seen in all seven horses during DN treatment. The ANOVA Test shows a trend to improvement (P = 0.044) in the right brachiocephalicus muscle. T Test also shows a statistical improvement (P = 0.013) on this side after 72 h post-puncturing. Statistics did not show improvement on the left side.

**Conclusions:** As the improvement on the right side is significant, if the sample size increased, a significant increase on the left side is likely to occur. The study shows that DN is an effective technique that provides proof of the existence of LTR when MtrPs are localised. Therefore, DN could be considered as a treatment for equine myofascial pain.

## A6 Treatment of the clinical symptoms caused by osteoarthritis using nuclear magnetic resonance (MBST^®^) in dogs a randomized trial—a pilot study

### Marion Mucha^1^, Ivonne Virac^1^, Cornelia Lang^1^, Kathleen Wittek^1^, Alexander Tichy^2^, Barbara Bockstahler^1^

#### ^1^Department for Small Animals and Horses, Small Animal Surgery, Section for Physical Therapy and Rehabilitation, University of Veterinary Medicine, 1210, Vienna, Austria, ^2^ Department for Biomedical Sciences, Platform Bioinformatics and Biostatistics, University of Veterinary Medicine, 1210, Vienna, Austria

##### **Correspondence:** Marion Mucha - marion.mucha@vetmeduni.ac.at

*Acta Veterinaria Scandinavica* 2016, **58(Suppl 2)**:A6

**Background:** Canine osteoarthritis (OA) is a commonly seen problem in veterinary practice. There are different methods available to treat related pain, stiffness and lameness. A recently developed method is the treatment with nuclear magnetic resonance (NMR). In this method a permanent magnetic field is combined with an interfering field.

**Objectives:** A double-blinded randomized trial was performed to evaluate if nuclear magnetic resonance treatment (MBST^®^) has a positive effect on the pre-treatment clinical signs of dogs suffering from osteoarthritis directly after the MBST^®^ treatment and 3 and 6 months after treatment.

30 dogs were included in the study. The inclusion criteria were a radiological confirmed osteoarthritis with clinical signs (pain during palpation and/or lameness) in the orthopedic examination.

Fifteen dogs received NMR treatment (TG) and 15 received a placebo (PG) over a period of nine days. To describe the overall clinical success of the MBST^®^ intervention the following parameters were evaluated: symmetry indices of peak vertical force and vertical impulse, lameness and pain score, drop-out, additional pain medication or physical therapy during the course of the study. From these parameters an individual score was calculated for each dog to evaluate the overall treatment effectiveness (OTE) at the evaluation points.

**Results:** In TG symmetry indices of vertical impulse and lameness score had significantly improved at 3 months after treatment. To compare the effect of the NMR treatment within the groups on the lameness and pain between day 0 and the subsequent measurements a Friedmann Test was used. To evaluate the changes in the GRF within the groups between day 0 and the subsequent measurements an ANOVA for repeated measurements was used. Differences in the overall score between groups were tested using a Mann–Whitney Test. P < 0.05 was considered statistically significant.

**Conclusions:** Findings from this study suggested that NMR had potential positive effects regarding the clinical signs of OA in dogs at 3 months after therapy.

## A7 Magnetic resonance imaging of sagittal plane translation of the human cervical spine during graded dorsal/ventral mobilizations and possible implications for dogs

### David Levine^1^, J. Randy Walker^1^, Āren Swogger ^2^, Tavis Gibson ^3^, Denis J Marcellin-Little^4^

#### ^1^Department of Physical Therapy, The University of Tennessee at Chattanooga, Chattanooga, TN, USA, ^2^Physical Therapy, Life Care Centers of America, Antioch, TN, USA, ^3^Star Physical Therapy, Clarksville, TN, USA, ^4^North Carolina State University, Raleigh, NC, USA

##### **Correspondence:** David Levine - david-levine@utc.edu

*Acta Veterinaria Scandinavica* 2016, **58(Suppl 2)**:A7

**Background:** Cervical dorsal to ventral (CDV) mobilizations are commonly used to treat pain and motion restrictions in the human cervical spine. These mobilizations may provide pain relief in dogs with cervical disease. Little is known about the motion of specific cervical vertebrae resulting from CDV mobilizations.

**Objectives:** The objective of this study was to determine the effects of graded CDVs to the dorsal spinous process of cervical vertebrae 2–7.

**Materials and methods:** Twenty healthy subjects (F = 14, M = 6) ranging from 21 to 32 years of age (median = 24), with no past cervical spine injury or pain in the last 6 months, and no contraindications for magnetic resonance imaging (MRI) or cervical mobilization were recruited. Subjects were positioned prone in an MRI scanner and spinal positions were scanned while a certified manual therapist performed grade II and III CDVs on each cervical vertebra (2–7). Appropriate hand placement on the spinous process was confirmed using MRI data prior to the mobilization. Sagittal plane cross sections were used to measure the position of each vertebra in the neutral position and during grade II and grade III CDVs. Movement of each vertebra was analyzed using image analysis software (OsiriX) to compare relative and absolute positions. Data were statistically analyzed using SPSS Version 19, (Armonk, NY).

**Fig. 5:**
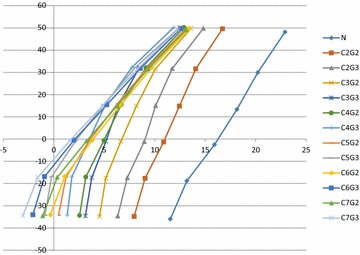
Cervical vertebra and grades. N = (neutral) represents the resting position of each vertebra prior to any mobilization. The seven points on the N plot are the seven cervical vertebra, the top is C2. This graph represents the absolute movement of each vertebra when a posterior to anterior (PA) mobilization (grade II or grade III) is applied. Absolute is defined as the change in position of a vertebra from its resting position when a mobilization is applied. The* horizontal axis* represents the amount of PA motion of each vertebra and* vertical axis* represents the amount of superior to inferior motion of the vertebra. A* negative number* represents a flexion moment while a* positive number* represents an extension moment

**Results:** All cervical vertebrae (C2–7) significantly changed from their resting position in the sagittal plane when grade II or III CDVs was performed on each cervical vertebra (*P* < 0.001; Fig. 5). Grade III CDVs produced greater anterior translation than grade II CDVs for each vertebra (*P* < 0.001). Grade II and III CDVs changed the position of each vertebra in relation to the vertebrae above and below (*P* < 0.001). Grade III CDVs on C7 resulted in the greatest relative translation of the lower cervical spine; grade III CDVs on C4 resulted in the greatest relative translation of the upper levels of the cervical spine. Grade III on C5 resulted in extension of C2–4 and extension of C6–7.

**Conclusions:** Direct dorsal to ventral mobilization to each cervical vertebra (grade II or III) changed the position of that vertebra and the position of all cervical vertebrae. The amount of displacement is proportional to the mobilization grade. Isolating motion to a single cervical vertebral segment with graded mobilization in the cervical spine does not appear possible; segments above extend, while segments below flex; C5 mobilization was an exception to this pattern.

## A8 Creation of an open-access Animal-Assisted Therapy exercise database using expert consensus with the Delphi Method: a pilot study

### David Levine^1^, Janice Ryan^1^, Chris Gilligan^1^, Katie Haulcomb^1^, Leigh Anne Norris^2^, Matt Powers^1^, Tracy Pugh^1^, Seth Purkey^1^

#### ^1^Department of Physical Therapy, The University of Tennessee at Chattanooga, Chattanooga, TN, USA, ^2^Siskin Hospital for Physical Rehabilitation, Chattanooga, TN, USA

##### **Correspondence:** David Levine - david-levine@utc.edu

*Acta Veterinaria Scandinavica* 2016, **58(Suppl 2)**:A8

**Background:** The American Veterinary Medical Association defines the human-animal bond as a mutually beneficial dynamic relationship between people and other animals that is influenced by behaviors that are essential to the health and well-being of both [1]. Utilizing the human-animal bond, animal-assisted therapy (AAT) is a specific type of rehabilitation therapy that involves using animals as a form of treatment for humans. The use of a pet during therapy may be viewed as less threatening, more engaging, and more exciting, which may improve the rapport between patient and therapist, improve participation, and enhance outcomes. The purpose of AAT is to improve an individual’s psychosocial and physical function, and is part of an individualized intervention delivered by a professional healthcare provider such as a physical or occupational therapist. In AAT the dog is utilized as an active partner in therapy, and is delivered as billable treatment.

**Objectives:** The purpose of this study was to create an open access AAT exercise database using expert consensus with the Delphi method.

**Materials and methods:** Exercises for inclusion were initially developed using multiple resources such as textbooks, journal articles, and expert consensus. Experts represented fields including physical therapy, occupational therapy, recreational therapy, and veterinary medicine. Exercises were developed using dogs, and with the aim of encompassing a large variety of exercises that could be useful in rehabilitation of a wide variety of physical impairments and disabilities. An example is brushing a dog to work on hand and finger grasp, fine motor skills, coordination, cognition, etc. Ten exercises were evaluated in this pilot study by experts to determine the search categories for each exercise (e.g. fine motor, gross motor, balance, cognition, range of motion, gait, etc.).

**Results:** Agreement between experts ranged from 38 to 100%. Exercises with agreement less than 70% were reviewed by an expert panel to determine a consensus, and to modify the description if needed.

**Conclusions:** The creation of this open access AAT exercise database using expert consensus is ongoing and will continue to evolve as exercises are added.

**Reference**

1. Human-Animal Bond. American Veterinary Medical Foundation. https://www.avma.org/kb/resources/reference/human-animal-bond/pages/human-animal-bond-avma.aspx.

## A9 Goniometric measurement of front limb rotation in labrador retrievers

### Hanna Pulkkinen, Anu Lappalainen, Outi Laitinen-Vapaavuori, Heli Hyytiäinen

#### Department of Equine and Small Animal Medicine, University of Helsinki, Helsinki, Finland

##### **Correspondence:** Hanna Pulkkinen - hanna.pulkkinen@helsinki.fi

*Acta Veterinaria Scandinavica* 2016, **58(Suppl 2)**:A9

**Background:** The goniometer is an objective and noninvasive tool for measuring joint positions in nonsedated animals. Measurement of front limb external rotation with goniometer in caninens has not yet been described.

**Objectives:** The study aimed to develop a novel protocol for measuring the rotational angle of the canine front limb with a goniometer.

**Materials and methods:** The study included 12 labrador retrievers, with a mean age of 3 ± 3 years and mean weight of 27 ± 4 kg. The rotation angle was measured in standing position with the goniometer’s axis under the metacarpal pad, the arms parallel to the spine and the 3rd digit. The measurements were repeated three times by two measurers, and the combined data of the two was used for descriptive statistics. Means were compared with paired t test, with significance at P < 0.05.

**Results:** The mean angle of external rotation was 15 °± 6° and the 95% confidence interval for the mean was 14–17°. There was no statistically significant difference between the left and right limb (P = 0.3) or between intra-tester measurements (P = 0.12). There was significant inter-tester variability (P = 0.005) with a mean difference of 3 °± 5° between measurers.

**Conclusions:** The study reported normal values for antebrachial rotation in healthy labrador retrievers. The good intra-tester reliability and statistical inter-tester difference is consistent with goniometric measurement studies in humans [1].

**Reference**

1. Lenssen AF, van Dam EM, Crijns YH, Verhey M, Geesink RJ, van den Brandt PA, de Bie RA. Reproducibility of goniometric measurement of the knee in the in-hospital phase following total knee arthroplasty. BMC Musculoskelet Disord. 2007; 17:83.

## A10 Test–retest reliability in a translated version of the Canine Brief Pain Inventory in canine osteoarthritis

### Ann Essner^1,2^, Rita Sjöström^3,4^, Lena Zetterberg^1^, Karin Hellström^1^, Pia Gustås^5^, Hans Högberg^6^

#### ^1^Department of Neuroscience, Uppsala University, Uppsala, Sweden, ^2^Evidensia Djurkliniken Gefle, Gävle, Sweden, ^3^Region Jämtland Härjedalen, Unit of Research Education and Development, Östersund, Sweden, ^4^Department of Community Medicine and Rehabilitation, Umeå University, Umeå, Sweden, ^5^Department of Clinical Sciences, Swedish University of Agricultural Sciences, Uppsala, Sweden, ^6^Department of Health and Caring Sciences, University of Gävle, Gävle, Sweden

##### **Correspondence:** Ann Essner - ann.essner@evidensia.se

*Acta Veterinaria Scandinavica* 2016, **58(Suppl 2)**:A10

**Background:** The Canine Brief Pain Inventory (CBPI) is a caregiver-reported questionnaire designed to capture pain severity and the impact of pain on activities in canine osteoarthritis (OA). The English version of CBPI has shown satisfactory intra-rater reliability. To be used repeatedly in outcome assessments the test–retest reliability of repeated caregiver-reported ratings with a translated version have to be properly tested. The hypothesis for testing the test–retest reliability was that there would be a strong relationship between two repeated caregiver-reported ratings.

**Objectives:** This study aimed to assess the test–retest reliability of a translated version of the CBPI (CBPI-S), in a group of dogs diagnosed with OA.

**Materials and methods:** Fifty-eight caregivers of dogs with OA were included in this cross-sectional study. Test–retest reliability was assessed by administrating the CBPI-S questionnaire twice at a 1 day interval. The relationship between the paired ordinal ratings was evaluated by Spearman’s ρ. The level of agreement was studied by a rank-based statistical method, the Svensson augmented ranking approach.

**Results:** Spearman’s rank-order correlation coefficients ranged from 0.86 to 0.92 in the CBPI-S total and sub-scores. All correlation coefficients were significant at the 0.01 level. The augmented ranking analysis revealed that there was no significant systematic disagreement present. However, the low nonzero value of the relative rank variance indicated presence of individual variability between the ratings.

**Conclusions:** The CBPI-S demonstrated strong to excellent correlations between repeated ratings. No systematic disagreement, but observed individual variability, indicated heterogeneity of the group. Our results confirm the hypothesis that the test–retest reliability of the CBPI-S is good in the group of dogs studied.

**Declarations:** The study was approved by the Local Animal Ethics Committee in Uppsala. Informed client consent was obtained for all animals used in the study. This study was funded by Sveland Foundation, Jan Skogsborg Foundation, and Agria Animal Insurance and the Swedish Kennel Club joint research fund. The authors have no competing interests to declare.

## A11 Validation of a Swedish version of the Helsinki Chronic Pain Index for the measure of chronic pain behaviors in canine osteoarthritis

### Ann Essner^1,2^, Anna Hielm-Björkman^3^, Hans Högberg^4^

#### ^1^Department of Neuroscience, Uppsala University, Uppsala, Sweden, ^2^Evidensia Djurkliniken Gefle, Gävle, Sweden, ^3^Department of Equine and Small Animal Medicine, University of Helsinki, Helsinki, Finland, ^4^Department of Health and Caring Sciences, University of Gävle, Gävle, Sweden

##### **Correspondence:** Ann Essner - ann.essner@evidensia.se

*Acta Veterinaria Scandinavica* 2016, **58(Suppl 2)**:A11

**Background:** Helsinki Chronic Pain Index (HCPI) is a caregiver-reported questionnaire designed to measure chronic pain behaviors in dogs with osteoarthritis (OA). The first Finnish version of HCPI has displayed satisfactory psychometric properties. To be used in another language the HCPI has to be properly translated and psychometrically tested.

**Objectives:** This study aimed was to report psychometric properties of a Swedish translation (HCPI-S) of the second version of HCPI.

**Materials and methods:** Twenty-one caregivers of clinically sound dogs and 58 caregivers of dogs with OA were prospectively included in this observational and cross-sectional study. After being translated into Swedish, according to recommendations for patient-reported outcome measures, the HCPI-S was completed by the caregivers. Construct validity was assessed by repeating the exploratory factor analysis by principal component model with subsequent varimax rotation and by assessing for differences between sound dogs and dogs with OA (extreme groups) using Mann–Whitney U test. Internal consistency was estimated by Cronbach’s α.

**Results:** Exploratory factor analysis showed a three-factor structure. The factors were extracted by showing eigenvalues >1. Together the factors accounted for 65.1% of the total variance. Clinically sound dogs showed significantly lower HCPI-S total score compared to OA dogs. Cronbach’s α was >0.7.

**Conclusions:** Our results supplement the knowledge with the HCPI by repeating satisfying construct validity and high internal consistency of HCPI-S in a group of dogs with OA.

**Declarations:** The study was approved by the Local Animal Ethics Committee in Uppsala. Informed client consent was obtained for all animals used in the study. This study was funded by Sveland Foundation, Jan Skogsborg Foundation, and Agria Animal Insurance and the Swedish Kennel Club joint research fund. The authors have no competing interests to declare.

## A12 Validity and reliability of 4Leg Check^®^—an instrument for outcome measuring the load distribution during standing in dogs

### Charlotte Orrfors^1,2^, Gunnevi Sundelin^1^

#### ^1^Department of Community Medicine and Rehabilitation Physiotherapy, Umeå University, Umeå, Sweden, ^2^Fysiohund Sverige, Stockholm, Sweden

##### **Correspondence:** Charlotte Orrfors - charlotte.orrfors@fysiohund.se

*Acta Veterinaria Scandinavica* 2016, **58(Suppl 2)**:A12

**Background:** Static weight distribution is considered to be a potential variable when lameness in dogs is evaluated. Instruments for this purpose, that are available in the market, lack research on their measurement properties.

**Objectives:** The objective was to assess the degree of validity and reliability of 4Leg Check^®^ during standing in dogs.

**Materials and methods:** 63 dogs participated. The scales criterion validity was controlled against control weights. The weight distribution was measured for all dogs with 4Leg Check^®^ during 3 s, three times in succession, followed by a manual assessment of the weight load of the fore and hind limbs pairwise. The relative reliability was calculated with Intraclass correlation coefficient (ICC) (2,1) and absolute reliability with Standard error of measurement (SEM and SEM%). Criterion validity, comparison of instrument and manual assessment, was calculated with Cohen’s kappa (κ).

**Results:** 4Leg Check^®^ showed 6.9–9.5 percent higher weight than control weights. The differences between weight sessions with control weights were consistent, ICC = 1.0. Relative reliability when measuring dogs repeatedly was little, if any, to moderate, ICC = 0.22–0.52. SEM ranged from ± 4.7–10.4 percent, corresponding SEM% 24.6–34.9 percent. Criterion validity was moderate, κ = 0.46–0.48.

**Conclusions:** The scales show good validity but regular calibration is recommended. Reliability between repeated measurements is very low to moderate with high measurement error. Further research, comparing 4Leg Check^®^ with the gold standard, such as force plate platform, or reliable and valid pressure walkway systems is recommended before the instrument can be classified as an evidence-based outcome measurement tool when measuring load distribution during standing in dogs.

## A13 Understanding the effect of individual characteristics on canine mobility: Dog Mobility Scale

### Luísa Gonçalves^1^, João Niza-Ribeiro^1,2^, Darryl L. Millis^3^, Augusto José de Matos^1,4^

#### ^1^Abel Salazar Institute for the Biomedical Sciences-ICBAS, University of Porto, Porto, Portugal, ^2^Department of Population Studies, ICBAS and Institute of Public Health (ISPUP), University of Porto, Porto, Portugal, ^3^Department of Small Animal Clinical Sciences, Canine Arthritis, Rehabilitation, Exercise and Sports Medicine (CARES) Section, University of Tennessee College of Veterinary Medicine, Knoxville, TN, USA, ^4^Animal Science and Study Centre/Food and Agrarian Sciences and Technologies Institute (CECA/ICETA), University of Porto, Porto, Portugal

##### **Correspondence:** Luísa Gonçalves - luisavgoncalves@gmail.com

*Acta Veterinaria Scandinavica* 2016, **58(Suppl 2)**:A13

**Background:** The understanding of dog movement or dog mobility is evolving, with particular emphasis on the study of the causes that might affect it. A Dog Mobility Scale (DMS) was developed and validated to assess mobility in dogs.

**Objectives:** The aim of this study was to analyse the relationship between individual characteristics of healthy dogs (size, body weight, height, breed, gender and body condition) and their mobility scores.

**Fig. 6:**
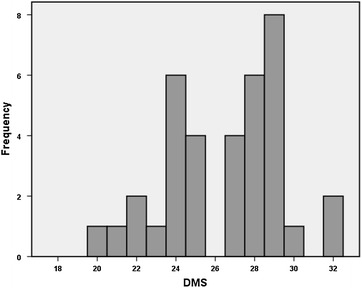
Histogram of the DMS frequency distribution

**Materials and methods:** Size, body weight, height, breed, gender, body condition score (BCS 1–9) and mobility score (DMS 0–32) (Fig. 6) were recorded from 36 healthy companion dogs. Statistical analysis of data included a descriptive analysis, the study of correlations using Pearson and Spearman coefficients (Table 2), the comparison of groups (Mann–Whitney test) and the analysis of variance (ANOVA). Significance level was set for P < 0.05.

**Table 2:** Pearson and Spearman correlation coefficients between age, gender, height, body weight, size, BCS and DMS of the 36 dogs

	Age (months)	Gender	Height (cm)	Body weight (kg)	Size	BCS	DMS
Age (months)							
*Pearson*	1.000	−0.160	−0.043	−0.005	0.009	0.268	−0.175
*Spearman*	1.000	0.003	0.160	0.244	0.230	*0.469* ^b^	0.016
Gender							
*Pearson*	−0.160	1.000	0.060	0.124	0.011	0.022	*0.375* ^a^
*Spearman*	0.003	1.000	0.027	0.090	0.017	0.044	*0.370* ^a^
Height (cm)							
*Pearson*	−0.043	0.060	1.000	*0.881* ^b^	*0.816* ^b^	0.051	−0.001
*Spearman*	0.160	0.027	1.000	*0.887* ^b^	*0.805* ^b^	0.086	−0.022
Body weight (kg)							
*Pearson*	−0.005	0.124	*0.881* ^b^	1.000	*0.932* ^b^	0.203	0.004
*Spearman*	0.244	0.090	*0.887* ^b^	1.000	*0.941* ^b^	0.240	−0.030
Size							
*Pearson*	0.009	0.011	*0.816* ^b^	*0.932* ^b^	1.000	0.143	−0.011
*Spearman*	0.230	0.017	*0.805* ^b^	*0.941* ^b^	1.000	0.200	0.027
BCS							
*Pearson*	0.268	0.022	0.051	0.203	0.143	1.000	0.022
*Spearman*	*0.469* ^b^	0.044	0.086	0.240	0.200	1.000	0.034
DMS							
*Pearson*	−0.175	*0.375* ^a^	−0.001	0.004	−0.011	0.022	1.000
*Spearman*	0.016	*0.370* ^a^	−0.022	−0.030	0.027	0.034	1.000

^a^Correlation is significant at the 0.05 level (2-tailed)

^b^Correlation is significant at the 0.01 level (2-tailed)

**Fig. 7:**
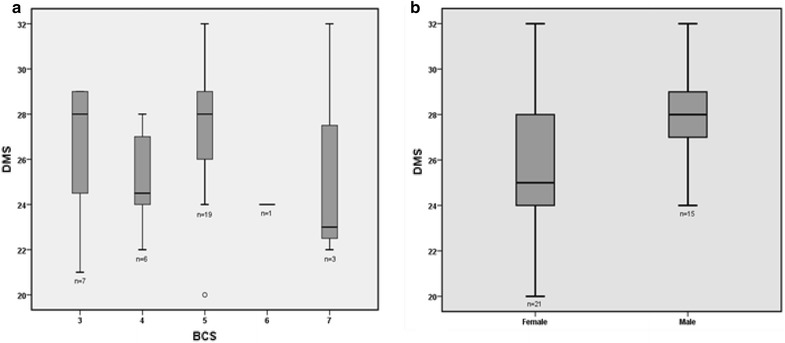
*Box*-and-*whisker plot* of the analysis of DMS according the BCS category (**a**) and gender (**b**)

**Results:** Dogs had an average (min–max) age of 35.25 months (3–216), BW of 18.61 kg (1.1–42) and height of 47 cm (21–70). Of the 36 dogs, 58.3% (n = 21) were female and 18 breeds were recorded, being 38.9% (n = 14) large, 27.8% (n = 10) medium, and 33.3% (n = 12) small breed dogs. Mean BCS was 4.6/9 and mean DMS score was 26.5/32. Males had statistically significant higher scores of mobility (Fig. 7), but with a weak correlation between the two variables. The remaining variables were not considered to affect mobility. As expected, a strong correlation was found between size, height and body weight (Table 2).

**Conclusions:** The mobility of healthy dogs is not affected by individual size, weight, height, breed or body condition, with the exception of gender. The results of this study enhance robustness of the instrument, as the DMS was developed to detect changes in mobility caused by orthopaedic or neurological pathologies. Further studies are needed to evaluate the DMS as an instrument for such purpose.

## A14 Comparative conventional, smartphone application and radiographic goniometric techniques of the equine metacarpophalangeal joint

### Marinette Teeling^1^, Kate Ross^1^, Victoria Geddes^1^, Ann Carstens^2^

#### ^1^Equine-Librium College, Plettenberg Bay, Western Cape, South Africa, ^2^University of Pretoria, Pretoria, South Africa

##### **Correspondence:** Marinette Teeling - marinette@equine-librium.co.za

*Acta Veterinaria Scandinavica* 2016, **58(Suppl 2)**:A14

**Background:** Physical therapy goniometry is the measurement of the angles through which a joint is able to flex and extend, used to quantify joint range of motion, decide on appropriate treatment and evaluate the effectiveness of treatment. The aim of this study is to validate a goniometric measurements using a smartphone application.

**Objectives:** Correlation of universal goniometer (UG), “DrGoniometer” (DrG) smartphone application (app) and radiographic measurements of metacarpophalangeal (MCP) angle.

**Materials and methods:** Metacarpophalangeal angle measurements of thirty horses were taken using a UG, the photography-based DrG app and a lateral digital radiograph. For the first readings a cast was used to standardize the angle of the MCP and in the second the MCP was in full flexion. The examiner holding the limb was blinded to the readings while the other recorded the UG values, and took the photograph and radiograph. Radiographic angles were measured and the positioning of the markers for DrG photograph angle measurements were determined. Pearsons’s correlations and ANOVA was performed on the data (P < 0.05).

**Results:** Flexed angles correlated well: DrG app versus radiographs showing the highest correlation (0.922), UG versus radiographic correlation 0.909 and UG versus DrG app 0.873. Cast correlations were less accurate: UG versus DrG 0.681, DrG versus radiograph 0.526 and radiograph versus UG 0.515. Significant angle differences were found between cast and flexed (P < 0.000), between cast UG and radiograph (P = 0.009), and no difference among the flexed angles.

**Conclusions:** Results indicate that the DrG app is a reliable tool to measure the angles of MCP flexion in the horse and appears to be more accurate than the conventionally used UG when compared to radiographs.

**Acknowledgements:** We thank Dr. Mike Ross and North Rand Animal Clinic for X-Ray machine usage and evaluation of radiographs, also the NRF South Africa for funding.

**Competing interests:** The authors declare that they have no competing interests.

a “DrGoniometer” (DrG) smartphone application, 31-33, rue Sainte Zithe, 2763, Luxembourg.

## A15 Determining hindquarter muscular and *tubera sacrale* asymmetry in training and racing Thoroughbreds

### Marinette Teeling^1^, Tineka Kriel^1^, Karien du Toit^1^, Jeanette Pauw^1^, Ann Carstens^2^

#### ^1^Equine-Librium College, Plettenberg Bay, Western Cape, South Africa, ^2^University of Pretoria, Pretoria, South Africa

##### **Correspondence:** Marinette Teeling - marinette@equine-librium.co.za

*Acta Veterinaria Scandinavica* 2016, **58(Suppl 2)**:A15

**Background:** Asymmetrical muscle development of the hindquarters of the racing Thoroughbred may result from one-leaded training and racing, possibly affecting future racing performance.

**Objectives:** To identify the incidence of hindquarter asymmetry (both muscular width and *tubera sacrale* height) in Thoroughbred racehorses in a stable where constant one-leaded training and racing was performed.

**Materials and methods:** The 25 Thoroughbred racehorses evaluated in this study were trained and raced on a left-leaded rein (clockwise). Caudal photographic images were taken of the hindquarters, while the horses stood square on a level cement surface. A transparent grid, placed on the screen of the camera was aligned in such a manner that the vertical line of the grid was directly aligned to the dorsal midline of the horse, while a horizontal line was aligned with the most dorsal part of the *tubera sacrale*. The images were printed and again a transparent grid was placed over them. Full and partially full grid blocks either side of the vertical line were counted to determine muscular asymmetry, and ventral to the horizontal line to determine *tubera sacrale* heights. The side on which the *tubera sacrale* was higher was termed the bony asymmetrical side, and the side with a larger muscular bulk (mostly the *musculus biceps femoris*) was termed the muscular asymmetrical side. Two authors (TK, AT) counted the grid blocks and compared their findings which were similar, but no inter- or intra-observer reliability was determined. An exact binomial test was performed on the data.

**Results:** Of the 25 horses, 21 of the horses (84%) had asymmetric hindquarters. Of these 21 horses, 16 horses (76%) showed significant muscular asymmetry having larger left hindquarter muscular bulk. This was significantly larger than the expected number given equal probability of left and right muscular asymmetry (P = 0.039). Twenty percent showed both muscular and bony asymmetry, and none only bony asymmetry.

**Conclusions:** It is suggested that training and racing to one side only, as in this stable’s case where the horses worked mainly on a left-leaded rein, can possibly lead to hindquarter muscular asymmetry that could affect racing performance.

**Competing interests:** The authors declare that they have no competing interests.

## A16 The effect of tendon boots on the skin temperature of the equine front limb

### Marinette Teeling^1^, Gillian Martindale^1^, Ann Carstens^2^

#### ^1^Equine-Librium College, Plettenberg Bay, Western Cape, South Africa, ^2^University of Pretoria, Pretoria, South Africa

##### **Correspondence:** Marinette Teeling - marinette@equine-librium.co.za

*Acta Veterinaria Scandinavica* 2016, **58(Suppl 2)**:A16

**Background:** Tendon fibroblast death has been reported to occur at 46 °C and since a six degree difference between skin and core temperature of the superficial digital flexor tendon (SDFT) in horses SDFT has been established [1], skin temperature can be used to approximate SDFT core temperature and can therefore assist in determining whether SDFT temperatures rise to injurious levels under certain perturbations.

**Objectives:** To determine palmar metacarpus three skin temperature (McTemp) of the equine forelimb on booted and non-booted limbs, at rest and post-exercise, and to determine rate of skin cooling after exercise.

**Materials and methods:** Part 1:18 Thoroughbreds’ McTemps were measured using a Benetech GM300 Infrared thermometer^a^ before and 1 h after stabling, with one forelimb booted and the other unbooted. Part 2: 14 Thoroughbreds’ McTemps were measured before and after 20 min lungeing at a trot, with one forelimb booted and the other bare; 1 min post cessation of exercise McTemp was again measured. Data were evaluated using paired t tests, with significance set at P < 0.05. No inter- or intra observer reliability evaluations were performed.

**Results:** The temperature measuring technique used has been reported to be reliable [1].

Average McTemp of non-booted and booted legs is given in Table 3.

**Table 3:** Average palmar metacarpal skin temperature in °C

Stabled (n = 18)				Exercised (n = 14)			
Non-booted		Booted		Non-booted		Booted	
Start	1 h	Start	1 h	Pre-	20 min post	Pre-	20 min post
26.7 ± 3.7	29.5 ± 2.5	26.2 ± 3.7	30.8 ± 1.8	25.7 ± 5.9	30.8 ± 5.3	25.4 ± 6.3	32.8 ± 5.3

The non-booted and booted legs cooled by 3–4 °C within 60 s of termination of exercise. The skin temperature of the booted leg was significantly higher than that of the non-booted legs both in the stable and immediately post-exercise (P < 0.001)

**Conclusions:** The average skin temperature of the booted legs (26.2 ± 3.7 °C stabled time 0 and 32.8 ± 5.3 °C 20 min post-exercise) was higher than non-booted (26.7 ± 3.7 °C stabled time 0 and 30.8 ± 5.3 °C 20 min post-exercise) but did not reach the 40 °C level which could be indicative of overheating of the SDFT.

**Competing interests:** The authors declare that they have no competing interests.

**Reference**

1. Wilson AM and Goodship AE. Exercised-induced hyperthermia as a possible mechanism for tendon degeneration. J Biomech. 1994; 27:899–905.

## A17 A radiographic study on the effect of hanging traction on the intervertebral disc space width in 17 Daschunds

### Kristine Mylo^1^, Sybrand S. van den Berg^1^, Marinette Teeling^1^, Ann Carstens^2^

#### ^1^Equine-Librium College, Plettenberg Bay, 6600, South Africa, ^2^University of Pretoria, Pretoria, 0110, South Africa

##### **Correspondence:** Marinette Teeling - marinette@equine-librium.co.za

*Acta Veterinaria Scandinavica* 2016, **58(Suppl 2)**:A17

**Background:** Hanging traction is a conservative technique to treat intervertebral disc herniation in chondrodystrophic dogs. This study was conducted using radiography to determine whether this form of treatment does in fact increase the intervertebral disc space (IVDS) width in Dachshunds. It was hypothesised that the IVDS width in sitting dogs (Si) would be equal to those in standing (St) positions and that those dogs in hanging positions (Ha) would have larger IVDS widths than those of both sitting and standing dogs.

**Materials and methods:** Seventeen healthy Dachshunds underwent lateromedial radiographs in sitting, standing and hanging positions. The most dorsal and most ventral aspects of the IVDS widths of T12/T13, T13/L1, L1/L2 and L2/L3 were measured three times, by two observers. The means of the measurements of Sit vs Std, Sit vs Ha and Std vs Ha were analysed using a student’s t test. (P < 0.05). There was no conflict of interest for any of the authors; all animals were treated according to the ethical requirements of the Equine–Librium College.

**Results:** There was good correlation between the measurements of the two observers (Table 4).

**Table 4:** Intervertebral space width (mm) and p values at each IVDS for each dog position

	Intervertebral space width (mm) and p values at each IVDS for each position (* significant difference)													
	Sit-stand					Sit-hang					Stand-hang			
	T12/T13	T13/L1	L1/L2	L2/L3		T12/T13	T13/L1	L1/L2	L2/L3		T12/T13	T13/L1	L1/L2	L2/L3
Dorsal														
si	2.16	2.35	2.43	2.44	si	2.16	2.35	2.44	2.44	st	2.15	2.42	2.58	2.58
st	2.15	2.42	2.58	2.58	ha	2.03	2.15	2.35	2.33	ha	2.03	2.15	2.35	2.33
Dorsal (P)	0.88	0.46	0.09	0.09		0.87	1	0.88	0.86		0.883	1	1	1
Ventral														
si	4.54	4.60	4.62	5.05	si	4.54	4.60	4.62	5.05	st	4.25	4.82	4.56	5.05
st	4.25	4.82	4.56	5.05	ha	4.43	4.55	4.59	4.98	ha	4.43	4.55	4.59	4.98
Ventral (P)	0.01*	0.58	0.53	0.92		0.87	0.75	0.65	0.75		0.04*	0.75	0.38	0.73

* There was no significant difference between any of the IVDS widths, except for the sit-stand and stand-hang IVDS widths at the ventral aspects of T12/T13

**Conclusions:** The fact that traction induced by hanging or sitting does not result in widened IVDSs in most of the positions, brings into question the supposed effectiveness of this form of conservative treatment. The statistically significant wider IVDS widths at the ventral aspect of T12/13 in the sitting and hanging positions, resulting in more hyperextension of the IVDS may be as result of this area being the most flexible part of the caudal thoracic area. More research is warranted to investigate this further.

## A18 The effect of the magnetic stimulation on the recovery time of canine thoracolumbar intervertebral disc disease

### Morito Ogasawara^1^, Hiromi Noguchi^1,2^, Takeo Minami^1,3^

#### ^1^Bayside Animal Clinic, Yokohama, Kanagawa, Japan, ^2^Shijonawate Gakuen University, Daito, Osaka, Japan, ^3^Minami Animal Hospital, Igaueno, Mie, Japan

##### **Correspondence:** Morito Ogasawara - ogasawara@minami-ahg.jp

*Acta Veterinaria Scandinavica* 2016, **58(Suppl 2)**:A18

**Background:** Magnetic stimulation is known as a non-invasive stimulation to excite and depolarize neurons in the brain and peripheral nervous system. Some studies indicate the magnetic stimulation may affect the progress of regeneration of the damaged spinal cord and neuroplasticity.

**Objectives:** This study is aimed to assess the effects of the magnetic stimulation on the recovery time of neurological conditions in dogs that had undergone surgery for thoracolumbar intervertebral disc disease.

**Materials and methods:** 33 dogs that had surgery for thoracolumbar intervertebral disc disease were included. 26 dogs as the not stimulated group included: 16 dogs with deep pain of hind limbs, and 10 dogs with no deep pain had a rehabilitation program once a week with hyperbaric oxygen therapy and underwater treadmill walking. 7 dogs in the stimulated group included: 4 dogs with deep pain, 3 dogs with no deep pain had the magnetic stimulation added to the rehabilitation program. MagPro R30 (MagVenture) with MMC-90 coil was used for the magnetic stimulation. Targets for the stimulation were damaged spinal cord region, sciatic nerve, femoral nerve, and sensory nerves on dogs’ pads. Recovery time of proprioception in hind limbs was assessed in each group. Mann–Whitney U test was used to compare the results.

**Fig. 8:**
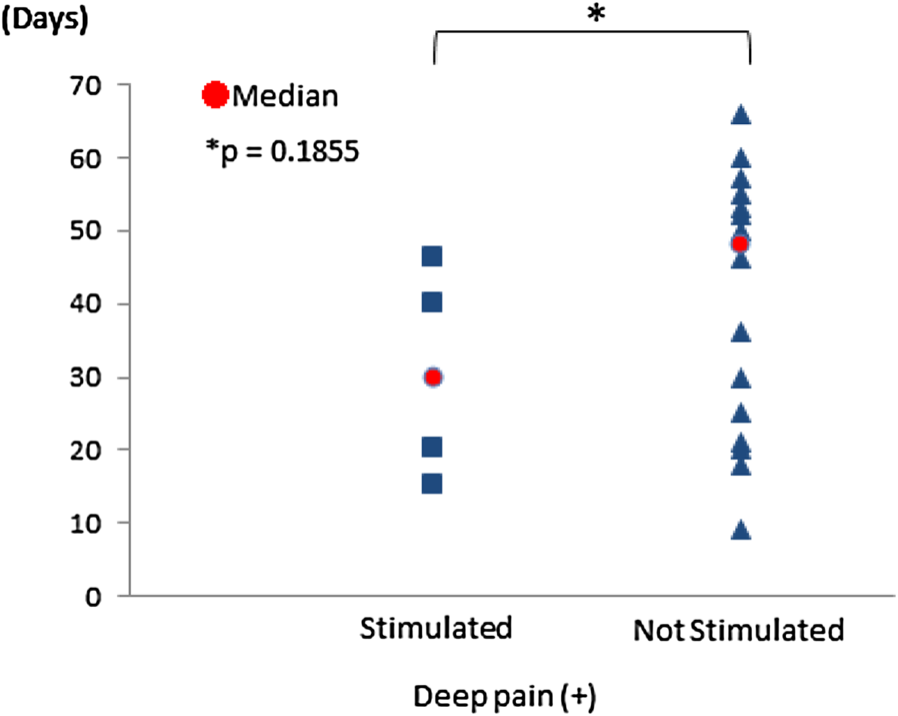
Recovering time of proprioception with deep pain present. The average (median) time to recover proprioception in stimulated and not stimulated group were 30.2 (30.0) and 42.1 (48.0) days, respectively

**Fig. 9:**
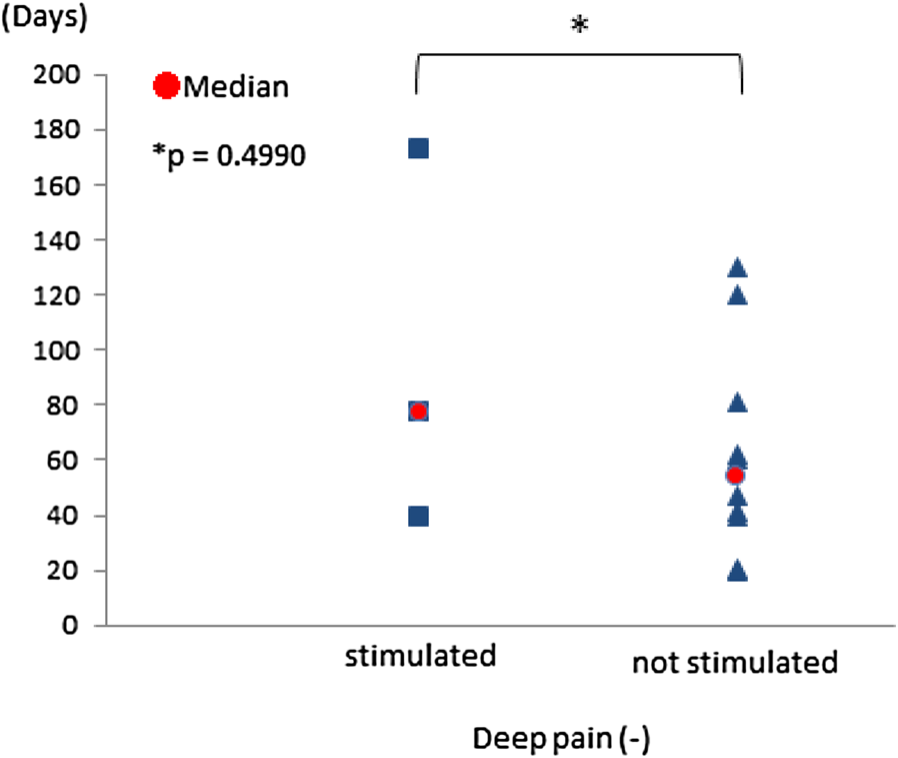
Recovering time of proprioception with no deep pain. The average (median) time to recover proprioception in stimulated and not stimulated group were 96 (77.0) and 62.2 (53.5) days, respectively

**Results:** The average (median) time to recover proprioception in stimulated and not stimulated group were 30.2 (30.0) and 42.1 (48.0) days with deep pain, 96 (77.0) and 62.2 (53.5) days with no deep pain, respectively. There was no significantly difference between stimulated group and not stimulated group in recovering time (Figs. 8, 9).

**Conclusions:** No significantly statistical difference between the stimulated group and not stimulated group existed in recovery time of proprioception, except for a shorter recovery time of proprioception in the stimulated dogs than the not stimulated dogs with deep pain present. Increased sample size is needed in further studies.

## A19 Rehabilitation programme for Great Dane with bionic hind limb prosthesis after partial amputation—case report

### Krzysztof Zdeb, Urszula Baumgart

#### Legwet Veterinary Clinic, Legionowo, Masovia, Poland

##### **Correspondence:** Krzysztof Zdeb - lecznica@legwet.pl

*Acta Veterinaria Scandinavica* 2016, **58(Suppl 2)**:A19

**Background:** A 6 year-old Great Dane female had undergone a partial hind limb amputation procedure due to osteosarcoma. Amputation was performed in the proximal third of the tibia. The titanium implant was introduced into the bone marrow cavity. The skin and muscles were attached to the implant.

**Objectives:** The objective was to achieve full weight bearing of the affected limb using a custom designed bionic prosthesis.

**Materials and methods:** The first goal was to minimize the muscle contraction caused by a two-week immobilization of the limb. Hot pack wraps were performed 2–3 times daily, followed by massaging of the thigh and stifle joint regions not involving skin of the operated region. The main interest was stretching contracted flexors and abductors of hip joint and flexors of the stifle joint. Afterwards, PROM exercises were introduced. Massage and PROM sessions were topped with passive extension 10 times for 30 s. The patient was then stimulated for weight bearing in standing position by pressing the temporary version of prosthesis to the implant and rocking the stern. This drove the dog to put the prosthesis on the ground. After obtaining correct positioning of operated limb, a four-stroke movement was introduced on the standard treadmill. All sessions were preceded by heat therapy, massage, and PROM. A few days of treadmill training showed great improvement in walking and controlled leash walks were introduced. In the same time, training of adaptation to normal functions like sitting, laying down, defecation, all using prosthesis were introduced.

**Results:** After achieving stable gait heat therapy and PROM exercises were reduced. Regular walk trainings were maintained. Clinical examination and X-rays performed a few months post operation showed stifle arthritis, likely caused by the lack of amortization from the hock. Kinematic gait analyze showed differences between the standing phase of the movement of operated and intact limb. The lack of hock joint gave the result in more flexed hip joint during the motion phase and extension of knee at the end of ground contact phase in the operated limb.

**Conclusions:** Partial limb amputation is a good procedure in dogs with malignant neoplasms. Using bionic bone-integrated prosthesis ensures stable gait and near-normal weight bearing. Lack of flexible hock causes discomfort in sitting and may lead to stifle arthritis due to little amortization.

## A20 Thoraco-lumbar disc herniation in a young male dog: orthopaedic complications due to hind limb loss of muscle mass

### Ana M. Ribeiro^1^, Ricardo Palas^1^, Martinho Capelão^2^

#### ^1^Physical Therapy Service, Pet Restelo Fisio & Spa, Restelo, 1400-195, Lisboa, Portugal, ^2^Neurology Service, Hospital Veterinário do Restelo, Restelo, 1400-195, Lisboa, Portugal

##### **Correspondence:** Ana M. Ribeiro - ana.mar.vet@gmail.com

*Acta Veterinaria Scandinavica* 2016, **58(Suppl 2)**:A20

**Background:** Neurologic conditions produce structural, chemical and biophysical alterations in the muscle [1–4] and tend to alter weight-bearing and limb use as a consequence of tissue atrophy [2, 4]. Rehabilitation plays an important role in improving strength, condition and function of those tissues [5–7].

**Fig. 10:**
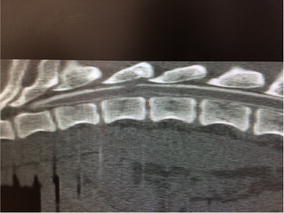
Computed tomography scanning image showing and myelography showing compression of the T11–T12 spinal segments

**Case description:** A 4-year-old intact male Golden Retriever with no past history of health problems presented to a Veterinary Hospital with hind limb paresis. After a computed tomography (CT) and myelography (Fig. 10), a Hensen II thoracolumbar Hernia was diagnosed between T11-T12. Because of the subtle nature of the lesion, medical treatment was chosen. During hospitalization, omeprazol, amoxicillin + clavulanic acid, enrofloxacin, robenacoxib were administered and cage rest was introduced. Three days after admission the dog regained function and proprioception of the left hind limb with no neurologic deficits but maintained no proprioception and diminished reflexes on the right hind limb. Ten days after initial presentation, the patient was discharged with oral medication and referenced to our rehabilitation centre.

**Fig. 11:**
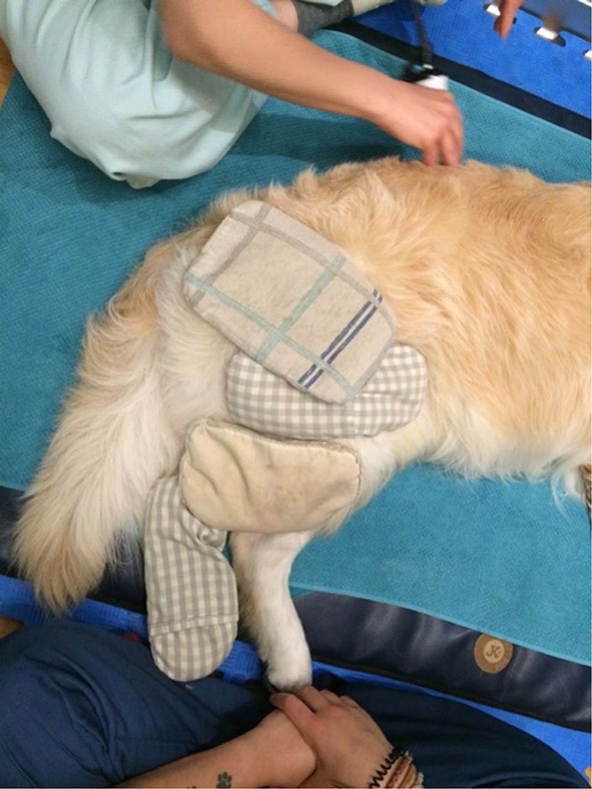
Thermotherapy with seed bags

**Fig. 12:**
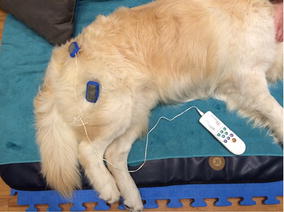
NMES of the hamstring muscles

**Fig. 13:**
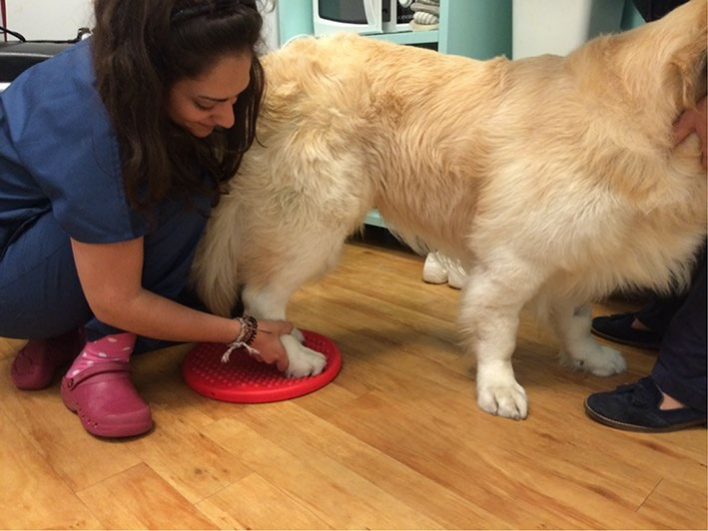
Right hind limb bicycle movements with balance disc

**Fig. 14:**
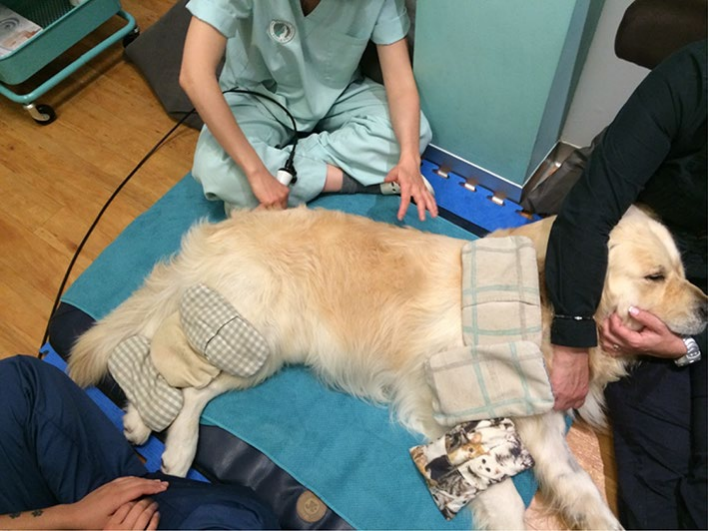
Laser therapy at lesion site (T11–T12)

**Fig. 15:**
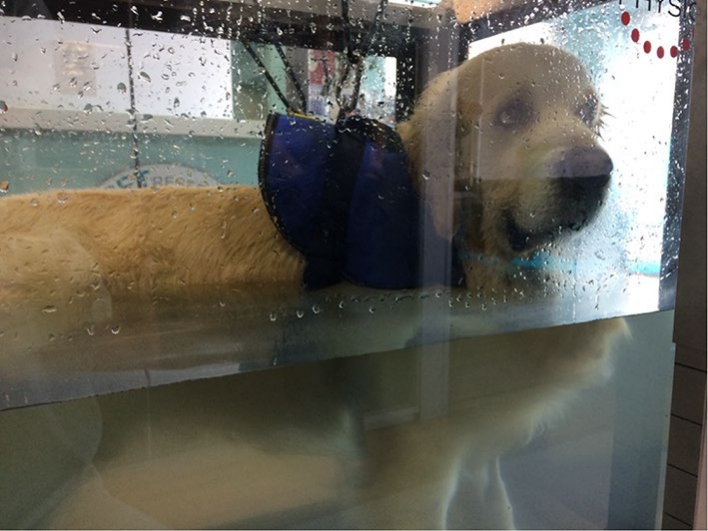
Underwater treadmill exercise

**Fig. 16:**
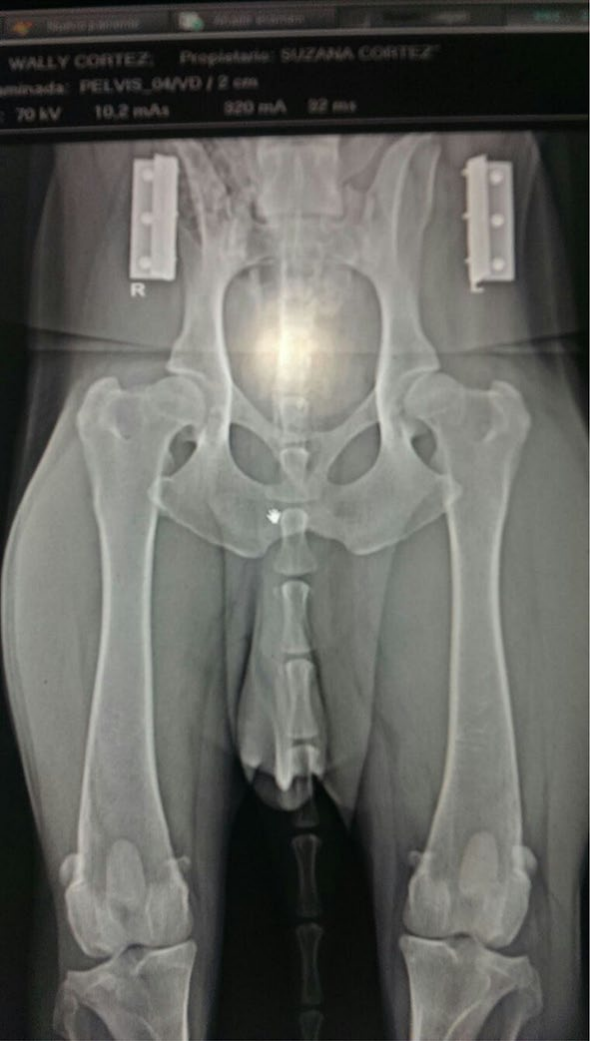
Radiological image of the hip joints in ventral-dorsal view showing grade D Hip Dysplasia

**Discussion:** At presentation the patient had no neurologic deficits on the left hind limb, reflexes were diminished and proprioception absent on the right hind limb, loss of muscle mass and strength was obvious. On the first week a daily rehabilitation protocol was initiated, including thermotherapy (Fig. 11), PROM, flexor reflex stimulation, neuromuscular stimulation (NMES) (Fig. 12), bicycle movements with balance disc (Fig. 13), laser therapy (Fig. 14) and 15 days after presentation hydrotherapy with underwater treadmill (UWTM) (Fig. 15) [7]. On the second week of treatment the sessions occurred every other day (EOD) and by the end of this week the patient had regained almost normal proprioception and reflexes, although loss of muscle mass was still marked. By the third week the treatment suffered a set-back when the patient became lame from the right hind limb with no neurologic alterations. An orthopaedic problem was suspected and the patient referenced to the orthopaedic service who made the diagnosis of Grade D bilateral Hip Dysplasia (Fig. 16).

**Conclusions:** Although the patient never had any symptoms or other signs of hip dysplasia, it is likely that the changes in the hip were pre-existing. The abrupt loss of muscle mass and strength that can occur after limb disuse [3–5] due to the paresis episode and can led to a unstable hip. Starting rehabilitation as soon as a neurologic injury occurs is the only way minimize muscle, bone and articular losses, that ultimately can lead to permanent alterations [5–7].

**Consent to publish:** Written informed consent was acquired from the person in Fig. 13.

**References**

1. Begun SJ, Reddy MM, Ramakrishna O, Indira K, Swami KS. Skeletal muscle protein metabolism under denervation atrophy in dog, *Canis domesticus*. Indian J Physiol Pharmacol. 1986; 30:341–6.

2. Brisson BA. Intervertebral disc disease in dogs. Vet Clin North Am Small Anim Pract. 2010; 40; 829–58.

3. Gordon T, Mao J. Muscle atrophy and procedures for training after spinal cord injury. Phys Ther. 1994; 74:50–60.

4. Millis DL, Levine D, Mynatt T, Weigel JP. Changes in muscle mass following transection of the cranial cruciate ligament and immediate stifle stabilization. In: Proc of the 27th Annual Conference of the Veterinary Orthopedic Society, Val d’Isere.

5. Millis DL. Responses of musculoskeletal tissues to disuse and remobilization. Canine rehabilitation and physical therapy. 2nd ed. 2014. pp. 93–153.

6. Monk ML, Preston CA, McGowan CM. Effects of early intensive postoperative physiotherapy on limb function after tibial plateau levelling osteotomy in dogs with deficiency of cranial cruciate ligament. Vet Res. 2006; 67:529–36.

7. Thomas WB, Olby N, Sharon L. Physical rehabilitation of the neurologic patient, Canine rehabilitation and physical therapy. 2nd ed. 2014. pp. 609–27.

## A21 Class IV laser therapy for treatment of deep dermatitis due to prolonged recumbency after knee surgery

### Ana M. Ribeiro, Ricardo Palas

#### Pet Restelo Fisio & Spa Restelo, 1400-195, Lisboa, Portugal

##### **Correspondence:** Ana M. Ribeiro - ana.mar.vet@gmail.com

*Acta Veterinaria Scandinavica* 2016, **58(Suppl 2)**:A21

**Background:** Laser therapy works by the application of electromagnetic radiation within the red and infrared spectrum over injuries and lesions to stimulate healing and pain relief through a process called photobiomodulation. Although its use continues to be controversial, Class IV Laser Therapy has been reported to be an adjunctive procedure for promoting healing of wounds through increased blood flow, release of growth factors, and by reducing inflammation without side-effects when properly used.

**Fig. 17:**
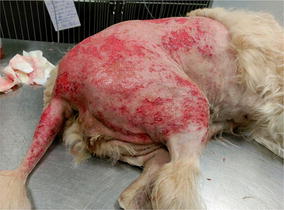
Day 0 before treatment

**Case description:** A 9 year-old neutered female canine underwent a cranial cruciate ligament surgery post rupture. One week after surgery the patient was referred for laser therapy treatment of deep dermatitis on the right hindlimb, due to prolonged recumbence in a surface with urine (Fig. 17). She had been treated with antibiotics (amoxicillin + clavulanic acid 12.5 mg/kg) and antiflammatory drugs (carprofen 4 mg/kg) for 8 days.

**Materials and methods:** Due to the painful nature of this lesion sedation with Propofol (4 mg/kg) was necessary. After sedation, the coat was trichotomized and the area cleaned with saline solution. For the laser therapy protocol a 10W class IV laser was used. The area to be treated was approximately 1000 cm^2^ and dosage was of 4 J/cm^2^. The time of treatment was 7 min with a total of four sessions, one session per day during 3 days and a last session 4 days later. No other topic treatments were administered.

**Fig. 18:**
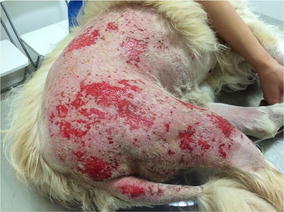
Day 1 after treatment

**Fig. 19:**
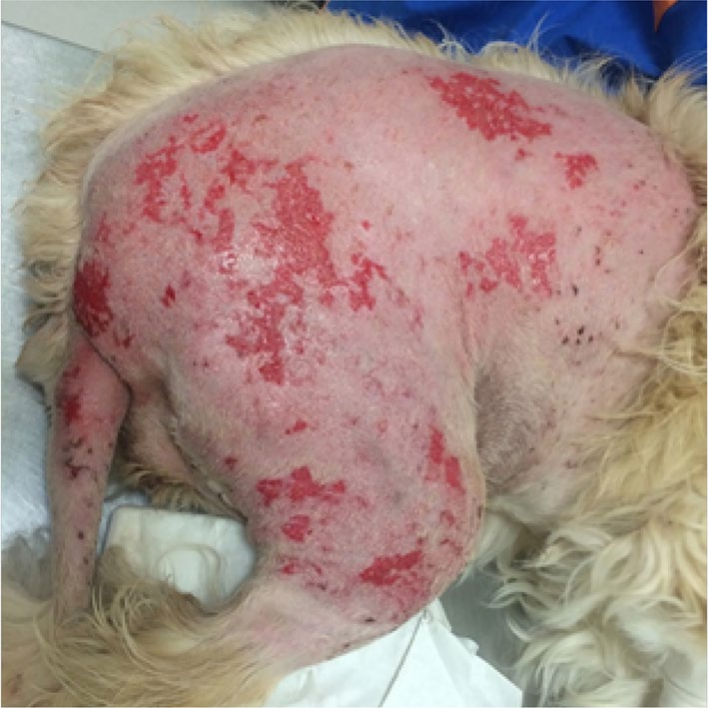
Day 2 after treatment

**Fig. 20:**
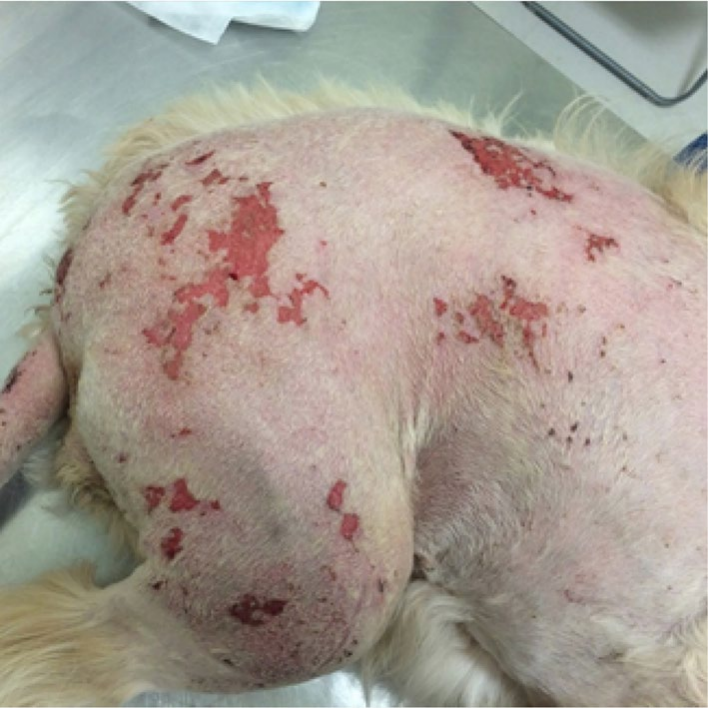
Day 3 after treatment

**Fig. 21:**
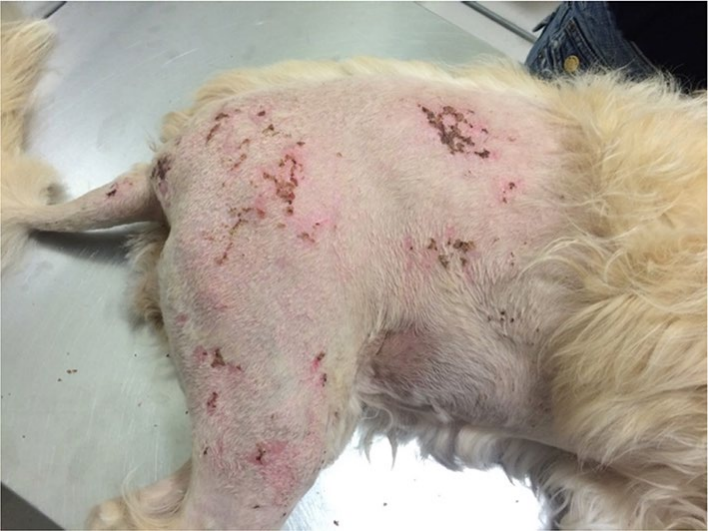
Day 7 after treatment

**Results:** After the first treatment inflammation and redness of the skin tissue visibly decreased (Fig. 18). The lesions were measured at every laser therapy session (Figs. 19, 20, 21). By the second session of laser therapy the patient was no longer in pain and manipulation was possible with no sedation needed. After 1 week (four sessions) of laser therapy the patient exhibited no signs of infection or inflammation and skin cicatrisation was nearly complete.

**Conclusions:** Considering tissue self-healing time, laser therapy is a useful treatment option for wound healing due to its easy usage, minimal time demands and rapid results without other topic pharmaceuticals or bandaging.

## A22 Clinical experience of postural and physical conditioning of the horse-rider unit

### Mila Speciani^1^, Alessandra De Luca^2^

#### ^1^Freelance Veterinarian, Mantova, Italy, ^2^KinesioProject asd, Bari, Italy

##### **Correspondence:** Mila Speciani - milaspeciani@gmail.com

*Acta Veterinaria Scandinavica* 2016, **58(Suppl 2)**:A22

**Background:** Both horse and rider rely on muscle chain activation to perform dynamic work, featuring different biomechanics patterns that become one functional unit when performing together.

**Objectives:** Aim of this study was to determine if horse’s and rider’s postural systems and biomechanical efficiency are reciprocally influenced.

**Fig. 22:**
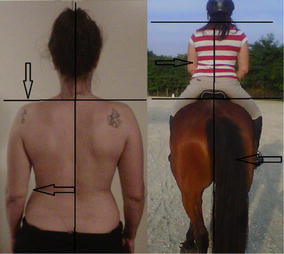
Rider’s postural asymmetries and horse-rider unit adaptation

**Fig. 23:**
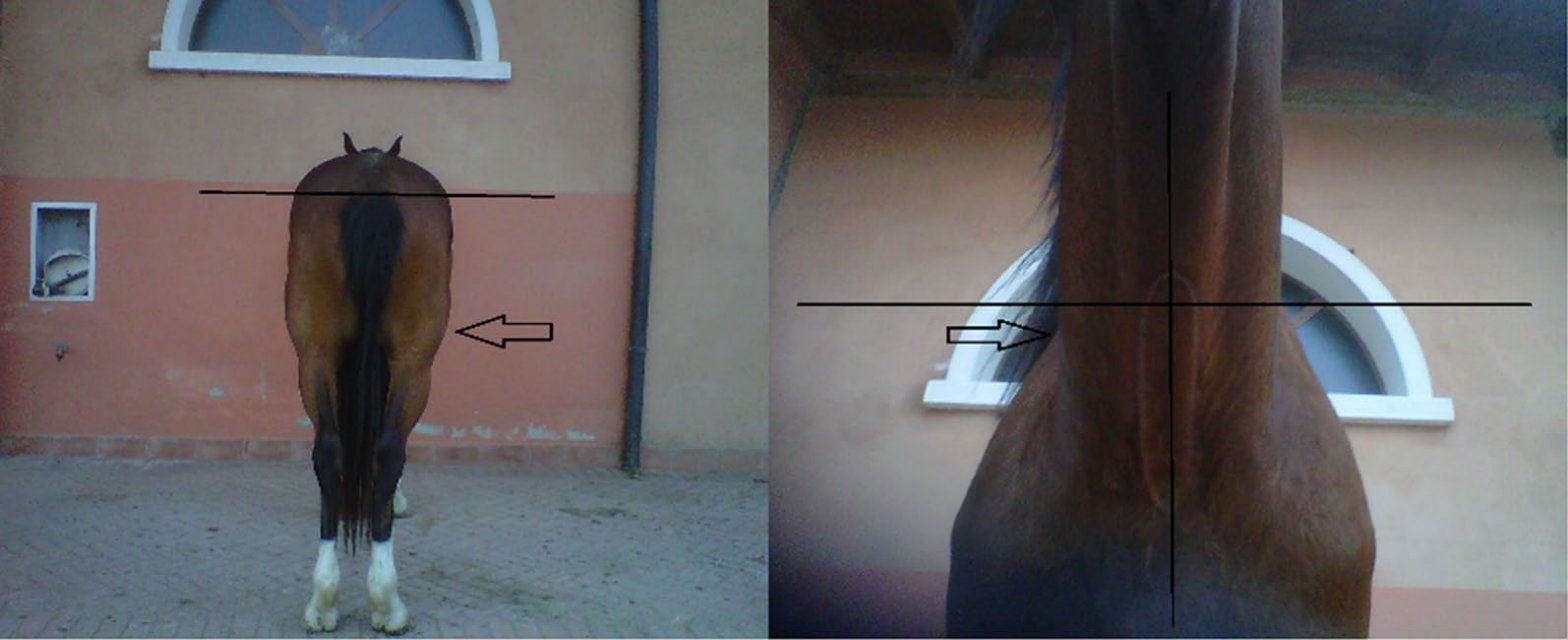
Horse’s muscling asymmetries due to compensation

**Fig. 24:**
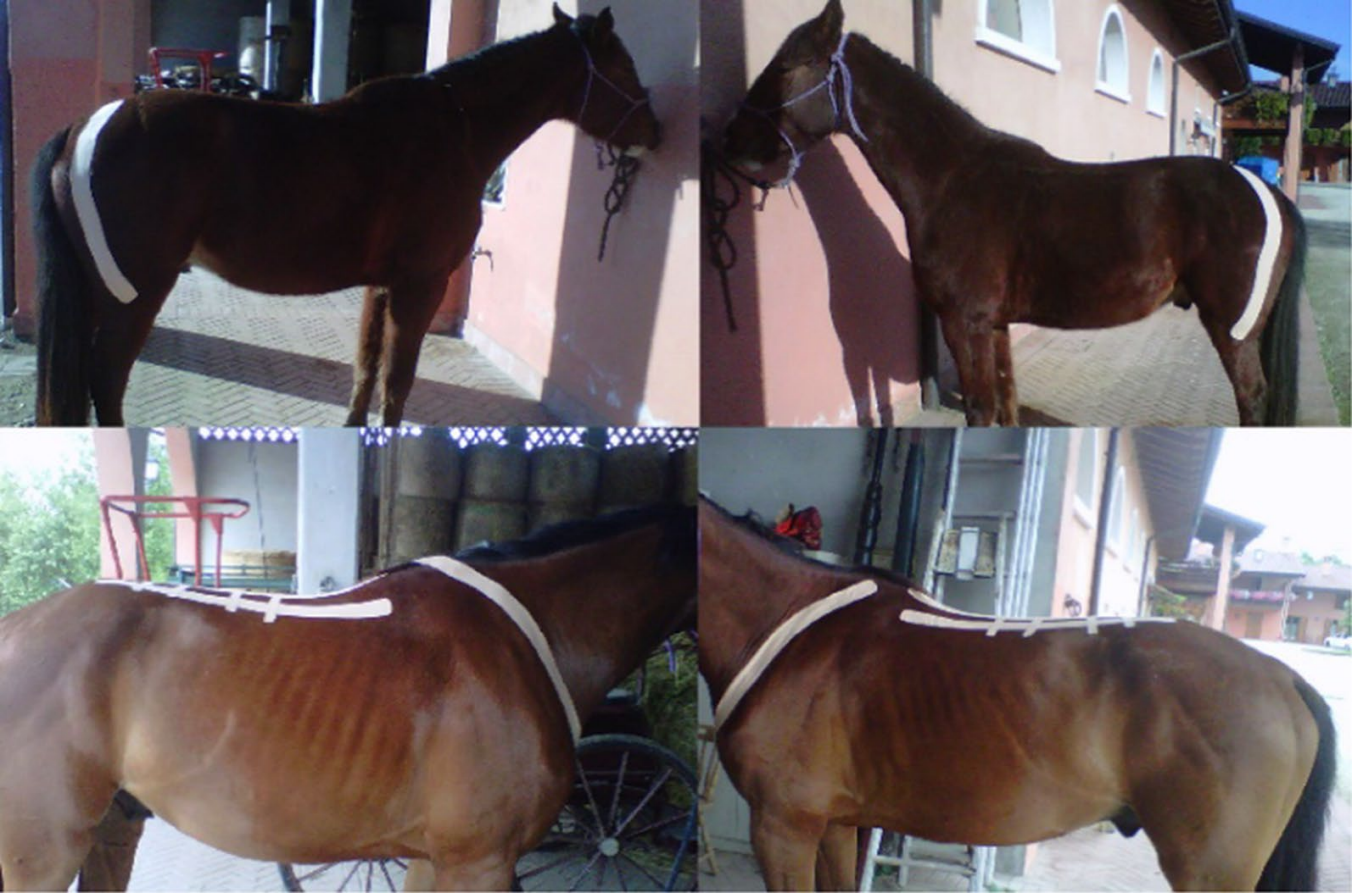
Kinesiotaping^®^

**Materials and methods:** We postulated that the more the unbalanced rider unevenly loaded the horse’s supporting muscle chains, the more the horse increased its own muscle load asymmetry, affecting movement dynamics of the horse-rider unit and further prompting rider’s unevenness on the saddle: uneven load on seat and stirrups, overall balance and comfort, effectiveness of aids. We developed postural re-education and conditioning plans for both athletes, balancing their individual biomechanics before reworking together. Our work included: (1) Postural evaluation of horse and rider individually and as horse-rider unit (Figs. 22, 23): muscular palpation, kinesiology muscle-testing, Fukuda test, Adams test, side-bending test, dental malocclusion check, ROM evaluation, visual documentation, white pad test, Borg Scale questionnaires administered to the rider. Postural analysis on the rider revealed a descending postural pattern due to dental malocclusion affecting muscle tone of several muscle chains (Fig. 22). Postural analysis findings on the horse included hypertonic left appendicular muscle groups, caused by compensation of inconsistent left-to right forces applied by the rider while on saddle, due to her muscular and postural asymmetries (Figs. 22, 23). (2) Active separation of the horse-rider unit by formulation of individual protocols for both athletes, aiming at neutralisation of reciprocal compensational mechanisms: myofascial release, bodywork, Kinesiotaping^®^ (Fig. 24) for the horse (1 session/week for 6 weeks, then monthly sessions); dental bite device, postural gymnastics and yoga for the rider (2 sessions/week for 8 weeks, then weekly sessions). (3) Active reunion of the horse-rider unit, performing postural conditioning exercises under saddle.

**Results:** Retests on rider after 4 months showed normotonic muscle chains, fatigue perception decrease and ROM increase. The horse also regained muscle symmetry and consistent bilateral muscle tone. Reintroducing mounted work and including maintenance sessions into their routine, both athletes maintained the acquired balance.

**Conclusions:** Horse and rider reciprocally affect their posture: treating and conditioning them both individually and as a whole, can improve and maintain comfort and effectiveness of the functional unit.

**Declarations:** The authors have written informed consent from the rider. This is available upon request.

## A23 Conservative multimodal approach (physical therapy and acupuncture) to a geriatric dog with spinal arthritis: a case study

### Mila Speciani^1^, Elisa Anzolin^2^

#### ^1^Freelance Veterinarian, Mantua, Italy, ^2^Ambulatorio Veterinario Anzolin, Verona, Italy

##### **Correspondence:** Mila Speciani - milaspeciani@gmail.com

*Acta Veterinaria Scandinavica* 2016, **58(Suppl 2)**:A23

**Background:** Integration of physical therapy and acupuncture for biomechanical issues and pain management shows increasing value in Veterinary Medicine. This combined approach has been chosen for treatment of a patient referred for PT and non-pharmacological management of his chronic spinal condition.

**Fig. 25:**
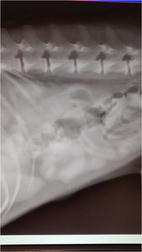
Radiographic findings (main lesion: L3–L4 osteophyte)

**Objectives:** The objective was to use a conservative holistic approach on a 14 year old male Bolognese dog, diagnosed with lumbar arthritis (main radiographic lesion: L3–L4 osteophyte) (Fig. 25).

**Fig. 26:**
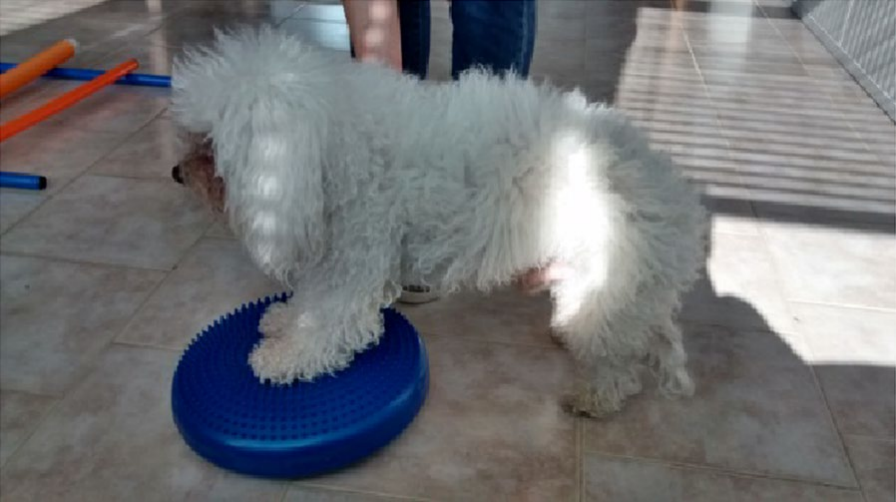
Patient performing exercise on a wobble pillow

**Materials and methods:** The patient received 10 weekly PT treatments integrated with 5 fortnightly acupuncture sessions, providing both biomechanical and energetic stimulation to control chronic pain and compensational postural issues. Initial findings included paraspinal muscles contracture and hind end muscle asymmetries. The patient was reluctant to voluntarily perform dynamic activities, showed kyphosis of lumbar-sacral region, fasciculation of right thigh muscles, uneven load of hind limbs. PT techniques applied during sessions included: myofascial release, pulsed-waves electromagnetic therapy, spike ball massage on muscle groups undergoing compensational mechanisms, dynamic gymnastics on poles, postural and proprioceptive exercises on a wobble pillow (Fig. 26).

**Fig. 27:**
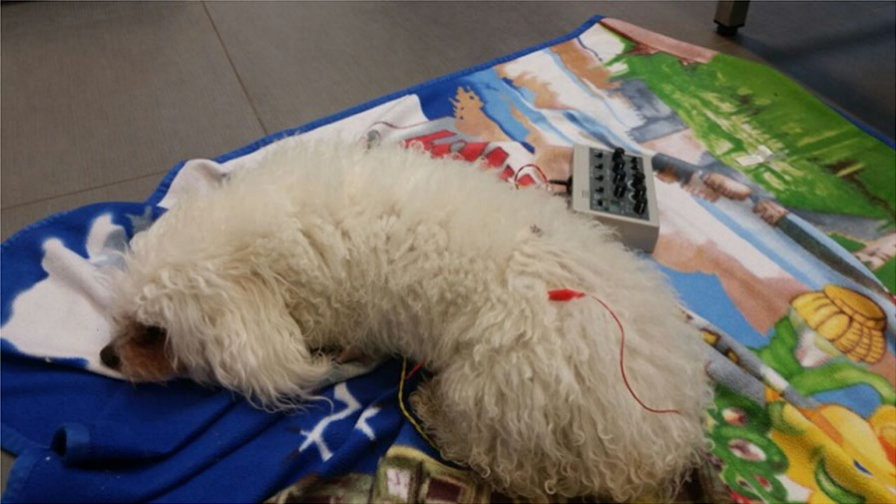
Patient receiving loco-regional electroacupuncture

Acupuncture treatments included: organic stimulation points (Spleen, Kidney, Small Intestine, Governor Vessel meridians) for energetic reactivation of the patient, and extraordinary meridians to balance the load of the hind limbs. Loco-regional electroacupuncture in the area of the osteophyte has also been applied (Fig. 27).

**Results:** Evaluations after the treatment cycle showed bilateral symmetry of appendicular muscles, and normotrophic/normotonic paraspinal muscle chains. Dynamic evaluation confirmed improvement of biomechanical efficiency, plus the dog demonstrated increased willingness to initiate movement. According to answers of pre- and post-treatment questionnaire administered to the owner, this patient elevated his life quality due to an improvement of his musculoskeletal condition (resolution of paraspinal muscle contracture and modification of thigh diameter from 21 cm left/25 cm right to bilateral 23 cm), supported by a higher level of energy and vitality (proper urination stance, increase in spontaneous physical activity, decrease of visible limb tremors, energetic and playful attitude). This dog continued maintenance sessions (PT every 20–30 days, acupuncture every 45–60 days) and followed up after 2 years without any relapse of symptoms.

**Conclusions:** The chosen conservative approach to this case study, integrating non-invasive techniques addressing both physical and overall emotional condition of the patient, allowed him to regain positive and interactive disposition towards physical activity.

## A24 Tail as an accelerometer attachment site when measuring foals’ motor behavior—a preliminary study

### Nina Pirinen^1^, Matti Pastell^2^, Anna Mykkänen^1^, Jonna Jokisalo^3^, Kati Niinistö^1^, Laura Hänninen^4^, Catherine McGowan^5^, Heli Hyytiäinen^1^

#### ^1^Department of Equine and Small Animal Medicine, Faculty of Veterinary Medicine, University of Helsinki, Helsinki, Finland, ^2^Natural Resources Institute of Finland (Luke), Green technology, Helsinki, Finland, ^3^Evidencia Hyvinkään Hevossairaala, Hyvinkää, Finland, ^4^Department of Production Animal Medicine, Faculty of Veterinary Medicine, University of Helsinki, Helsinki, Finland, ^5^Department of Musculoskeletal Biology, Institute of ageing and chronic disease, University of Liverpool, Liverpool, UK

##### **Correspondence:** Nina Pirinen - nina.pirinen@helsinki.fi

*Acta Veterinaria Scandinavica* 2016, **58(Suppl 2)**:A24

**Background:** Three dimension accelerometers (3DA) have been validated for evaluating adult horses’ behavior and locomotion [1, 2]. However, there are no reports of using 3DAs for collecting information on foals. In adult horses the accelerometers were attached to a limb, which is not safe for a long-term observation of foals. We aimed to study the usability of 3DA attached to foals tails to quantify their gross motor behavior.

**Materials and methods:** Two and four day-old male Warmblood foals, patients at the Helsinki University Veterinary Teaching Hospital, participated in the study for 3 and 12 days, respectively. A 3DA (HOBO Pendant H Data Logger, Onset Computer Corporation, USA) was attached to the base of each foal’s tail with positive Z-axis pointing cranially, positive X-axis vertically, and positive Y-axis laterally to the right. Raw acceleration was logged every 10 s with measurement range of ±3 g. Continuous video recordings (MS-C2163-PN, Milesight, USA) 20 frames/second of foals were taken. The start and end time of 20 occurrences of each behavior was recorded from both foals from video and combined with the corresponding times from the raw accelerometer data. This yielded a total of 5587 acceleration samples. The absolute difference between total acceleration and the gravity component [(Equation)] was used to calculate a movement index at each point. The position of the accelerometer from each axis and the movement index were compared between different behaviors using a pairwise Wilcoxon test with Holm correction (R 3.30).

**Fig. 28:**
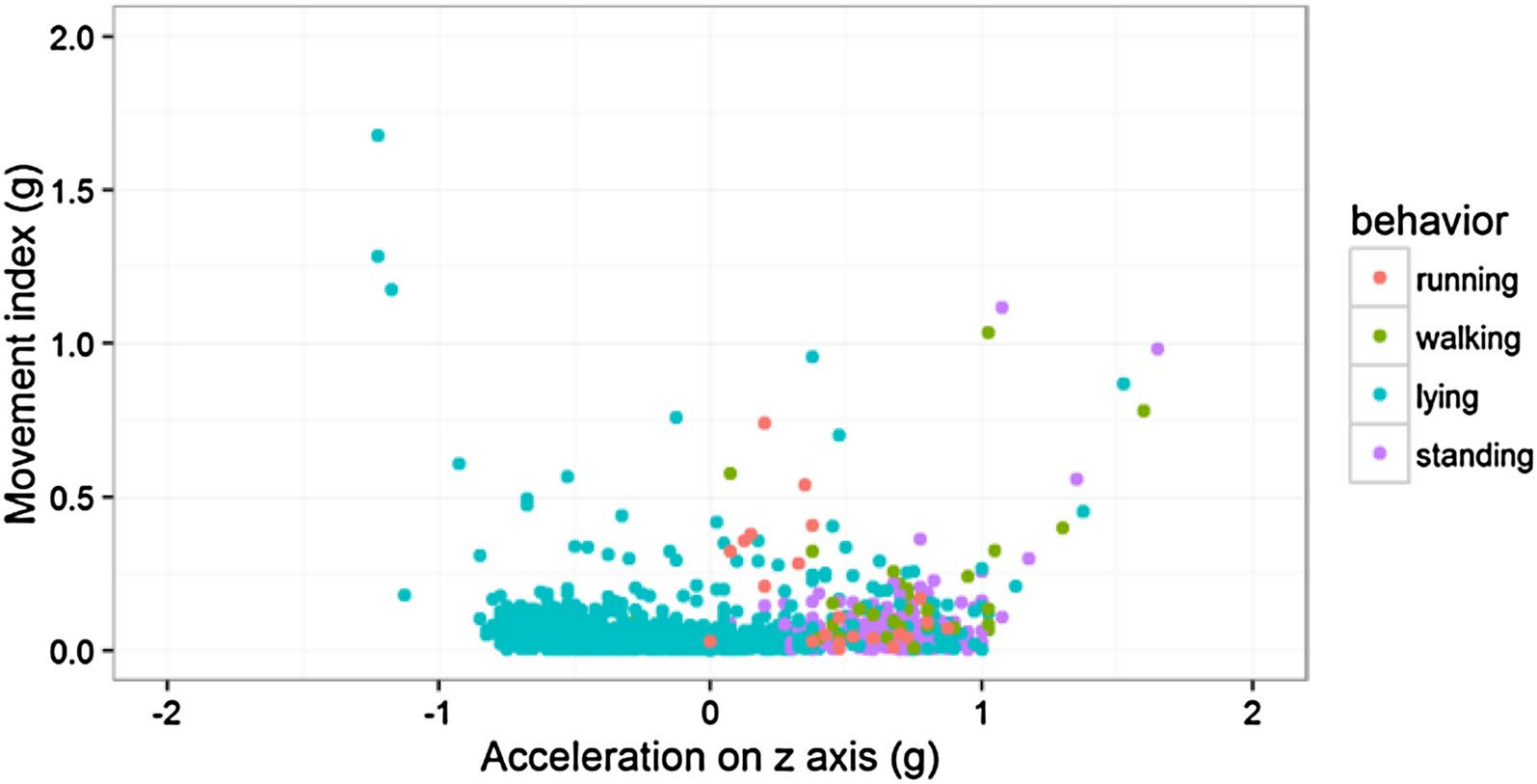
The acceleration on the* z-axis* and the motion index values for different behaviors and each data point

**Results:** The acceleration on the z-axis differed between all behaviors (P < 0.001). The measured (median ± SE in *g*) acceleration values, were 0.45 ± 0.08 running, 0.725 ± 0.08 walking, 0.675 ± 0.01 standing, and −0.375 ± 0.003 lying. The movement index differed between all other behaviors except running and walking. The measured (median ± SE in *g*) motion index values were 0.08 ± 0.04 running, 0.10 ± 0.03 walking, 0.05 ± 0.006 standing, and 0.03 ± 0.001 lying, data shown in (Fig. 28).

**Conclusions:** Behaviors such as lying, walking and running can be differentiated using a 3DA attached to a foals tail. However, further studies on the subject are warranted; data from more foals is needed to develop an automatic classifier and more informative results about dynamic motion could be gained using a higher sampling rate.

**Declarations:** The study protocol was approved by the University of Helsinki Viikki Campus Research Ethics Committee, and written consent was obtained from all owners. The study was funded by Emil Aaltonen’s foundation.

**References**

1. DuBois C, Zakraisek E, Haley DB, Merkies K. Validation of triaxial accelerometers to measure the lying behavior of adult domestic horses. Animal. 2015; 110–4.

2. Burla JB, Ostertag A, Schultze Westerath H, Hillman E. Gait determination and activity measurement in horses using an accelerometer. Comput Electron Agric. 2014;127–33.

## A25 Systematic review of the pathophysiology and management of canine patella luxation

### Alexandria Holt^1^, David Levine^1^, Denis J. Marcellin-Little^2^

#### ^1^Department of Physical Therapy, The University of Tennessee at Chattanooga, Chattanooga, TN, USA, ^2^Department of Clinical Sciences, North Carolina State University, Raleigh, NC, 27607, USA

##### **Correspondence:** David Levine - david-levine@utc.edu

*Acta Veterinaria Scandinavica* 2016, **58(Suppl 2)**:A25

**Background:** Patella luxation (PL) is one of the most common orthopedic problems in dogs, occurring mostly as a consequence of developmental orthopedic diseases and trauma. Patellar luxation has been managed conservatively and via surgical procedures. The pathophysiology and management of patellar luxation has not been systematically reviewed.

**Objectives:** The purpose of this systematic review was to identify and evaluate the evidence regarding the pathophysiology and management of canine patellar luxation by use of the FDA’s evidence-based medicine scoring system.

**Materials and methods:** A bibliographic search of online databases was performed through January 2016. Search terms included canine, dog, knee, stifle, dislocation, luxation, injuries, patella, patellar, patella and patellar dislocation, patella and patellar luxation, and Salvati. Articles that described the etiology, pathophysiology, diagnosis, management, and prognosis of patellar luxation in dogs were eligible for inclusion. Articles written in languages other than English were excluded. Articles describing patellar luxation in species other than dogs were excluded. The analysis consisted of study design rating, consistency rating, and cumulative strength of evidence ranking. Articles were then categorized based on their content and levels of evidence. Levels of evidence were graded using the Oxford Centre for Evidence-based Medicine scale. Articles were ranked under one or more of the following categories: etiology, pathophysiology, diagnosis, nonsurgical management, surgical management, and/or prognosis. Each category was graded on a scale of I (systematic review) through V (expert opinion). Reviews and prospective studies received lower numbered grades. Retrospective, case-series, and expert opinions received higher numbered grades.

**Results:** 301 studies were identified. 222 were excluded because the articles were not written in English (N = 11), involved species other than dogs (N = 21), were unrelated to patellar luxation in dogs (N = 188), or were not peer-reviewed (N = 2). Seventy-nine studies met the inclusion criteria. 27 total articles describing the pathophysiology of canine patellar luxation received grade II (N = 18), IV (5), or V (4). Fifty-nine articles describing the management/outcomes/complications of canine patellar luxation received grade II (N = 8), III (1), IV (32), or V (18).

**Conclusions:** With fewer than 20 experimental studies, little is known overall about the pathophysiology of patellar luxation. Fewer than 10 studies assessed the management of patellar luxation prospectively. Majority of the studies describing the management of patellar luxation are retrospective (54%) or opinion-based (31%).

## A26 Lameness management in a working dog with bilateral metacarpal lesions and sesamoidal stress fractures

### Mila Speciani

#### Freelance Veterinarian, Mantua, Italy

##### **Correspondence:** Mila Speciani - milaspeciani@gmail.com

*Acta Veterinaria Scandinavica* 2016, **58(Suppl 2)**:A26

**Fig. 29:**
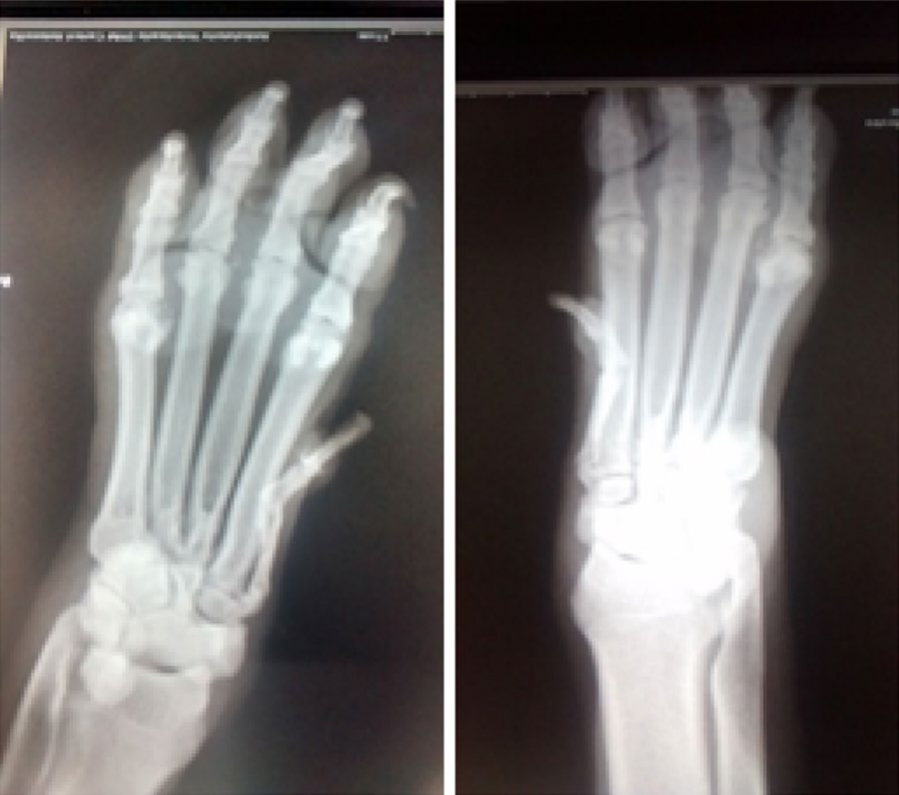
Initial radiographic findings

**Background:** A 3 year-old male working Labrador (active at the local public utility K9 unit) with a recent history of 2-months front limb shifting-leg 2/5 lameness and with radiographic findings of bilateral sesamoidal fragmentation and distal metacarpal erosion (Fig. 29), was referred for physical therapy.

**Objectives:** The goal for this clinical case was to exactly identify and consequently treat the biomechanical issues that caused the bilateral stress lesions in order to solve the lameness and allow the dog back to return to work after a proper reconditioning program.

**Materials and methods:** The approach to this patient included:

1. Initial static and dynamic evaluations and identification of the underlying biomechanical patterns that likely caused bone lesions and lameness. The dog showed the tendency of loading his front end more than his hind end (bilateral shoulder and pectoral muscles showing relative hypertrophy and hypertone); there was uneven left-to-right hind limb load; and varus deformity (Fig. 30).

**Fig. 30:**
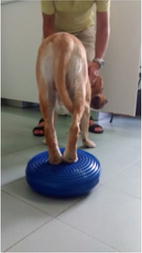
Caudal view of the patient on a wobble pillow
2. A cycle of 10 weekly physical therapy sessions including spike ball massage, pulsed-waves electromagnetic therapy (MaRhyThe^®^Matrix-Rhythmus-Therapie^®^), postural, wobble pillow (Fig. 31) and poles exercises, mainly aimed at postural correction, through reinforcement of paraspinal and hind limbs muscle chains, and relaxation of the anterior muscle groups, therefore prompting a shift of weight load onto the hind end.

**Fig. 31:**
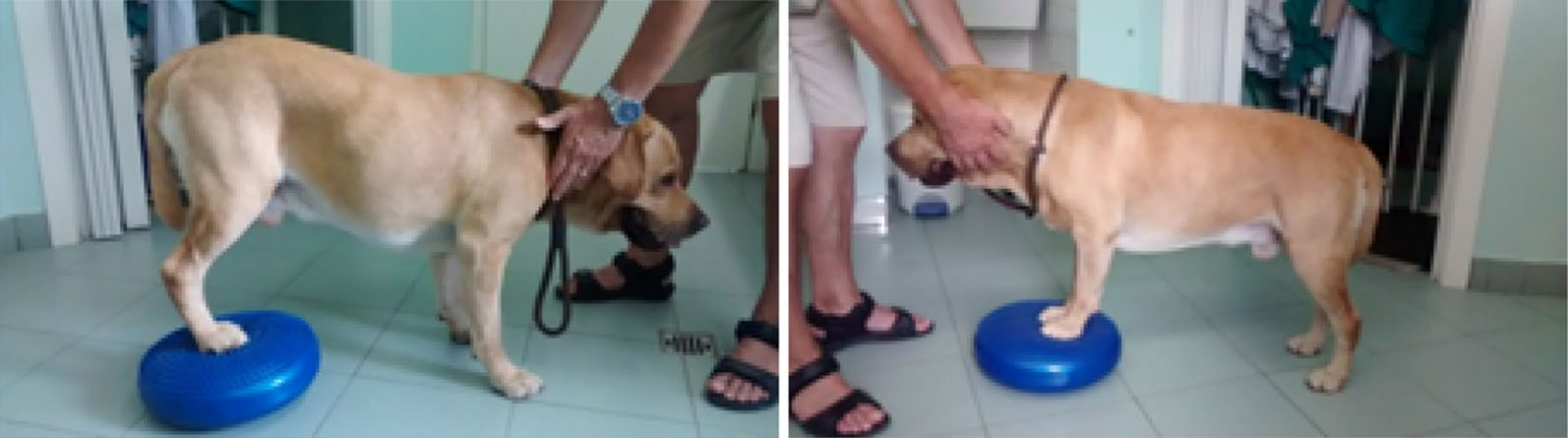
Patient performing exercises on a wobble pillow
3. In between sessions the dog followed a specific schedule of exercises (e.g. sit-to-stand and forward pulls) as well as a progressive reconditioning program, as the lameness grade consistently decreased.

**Results:** Along the treatment period, the patient constantly improved, lameness completely disappeared around the 3rd week of treatment, and then the dog was ready to progressively increase his conditioning program in order to go back to his regular training and working schedule. He went back to work at full regimen shortly after the 10-weeks treatment cycle, regularly receiving maintenance physical therapy treatments (monthly sessions, decreasing to one session every 45–60 days).

**Fig. 32:**
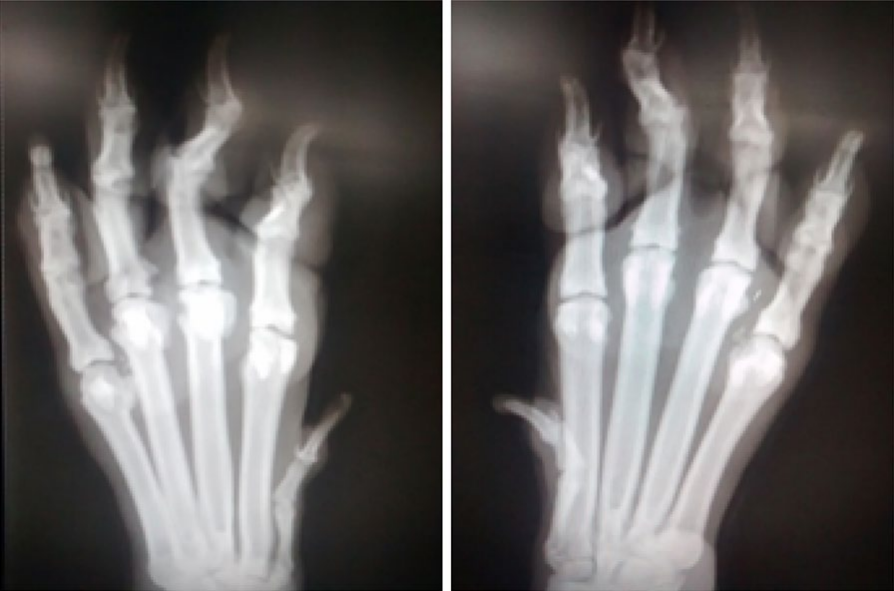
Radiographic findings after 1 year of follow up

**Conclusions:** After 1 year of soundness, regular work and maintenance treatment sessions, X-rays still show the same lesions as at day zero (Fig. 32), confirming that postural balance, proper conditioning and physical therapy aiming at control of conformational, compensational and biomechanical issues play a very relevant role for the effectiveness and comfort of movement.

## A27 Physiotherapy for paraplegic deep pain negative patients: development of spinal walking

### Marta Subirats^1^, Maria Perez^2^

#### ^1^Small Animals Physiotherapy Service, Firvet Fisioterapia y Rehabilitación Veterinaria, Polinyà, Barcelona, 08213, Spain, ^2^Department of Clinical Science Neurology/Neurosurgery Service, College of Veterinary Medicine, Mississippi State University, Mississippi, 39760, USA

##### **Correspondence:** Marta Subirats - martafirvet@gmail.com

*Acta Veterinaria Scandinavica* 2016, **58(Suppl 2)**:A27

**Background:** Patients with complete spinal cord section at T3–L3 segments can develop spinal walking as a result from intricate dynamic interactions between a central program in the lower thoracolumbar spine and proprioceptive feedback from the hind limbs. Thanks to automatism developed at birth and crossed extensor reflex work, paraplegic deep pain negative patients can recover motion of the hind limbs by stimulation of hind limb reflexes. A specific training program is suggested to be performed along with the development of spinal walking in dogs and cats after a severe spinal injury.

**Objectives:** To be an informative study without data collection and show the type of physical therapy we have been performing in paraplegic deep pain negative patients to help the development of a more controlled automatic gait or spinal walking. These animals will never recover deep pain sensation but do regain ambulation (with or without support), throughout a specific training and development of spinal walking.

**Materials and methods:** This protocol was established in 15 small dogs (1–5 kg) with completed spinal cord sections between T3 and L3. These dogs were administered the same treatment plan, with the same number and duration of sessions. The proposed program lasts 6 months. It consists in repeated underwater treadmill and land treadmill exercises, proprioception training, and passive and active therapy. A 20% increase in weekly exercise load, with an end goal of 20 min sessions of underwater treadmill or land treadmill exercises, plus 15 min of active and proprioceptive exercises and, the 25 min of passive therapy have been performed in our patients arriving to our suggested sessions of 1 h three times a week. The use of a harness or wheelchair may be recommended for support during the sessions to maintain the correct standing position to be able to work properly. In-house sessions are combined with home exercises.

**Results:** Most of the patients that received this specific protocol developed a spinal walking within 6 months. The patients re-learned the ability to walk in a straight line without losing their balance with or without the support of a wheelchair or harness for long walks, especially running, playing, or changing directions.

**Conclusions:** Paraplegic deep pain negative patients with lesions localized between T3 and L3 segments receiving a specific rehabilitation program can develop spinal walking within 6 months. Further data collection is recommended for future study to obtain repetitive results and demonstrable benefits.

## A28 *Rectus abdominis* functional test for measuring muscle force and fatigue resistance with EMG in horses

### Tatiana Hernández^1^, Luna Gutierrez-Cepeda^2^, Rafael Cediel^2^, Javier López-San Román^2^

#### ^1^Fisioveterinaria, 28523, Madrid, Spain, ^2^Universidad Complutense de Madrid, 28040, Madrid, Spain

##### **Correspondence:** Tatiana Hernández - luna.gutierrez@vet.ucm.es

*Acta Veterinaria Scandinavica* 2016, **58(Suppl 2)**:A28

**Background:** Therapeutic exercises have been proposed when planning a rehabilitation protocol [1]. However, few specific exercises have been reported to produce a functional improvement in horses [2, 3]. In order to progress on the design of effective rehabilitation and conditioning plans, these exercises need to be assessed with objective methods in terms of muscle force and fatigue. Superficial electromyography (EMG) has demonstrated to be useful for this purpose in human research [4] but this type of tests needs to be standardized for horses.

**Objectives:** To describe, prove and discuss three different protocols, using EMG as an objective functional test to measure muscle force and fatigue.

**Fig. 33:**
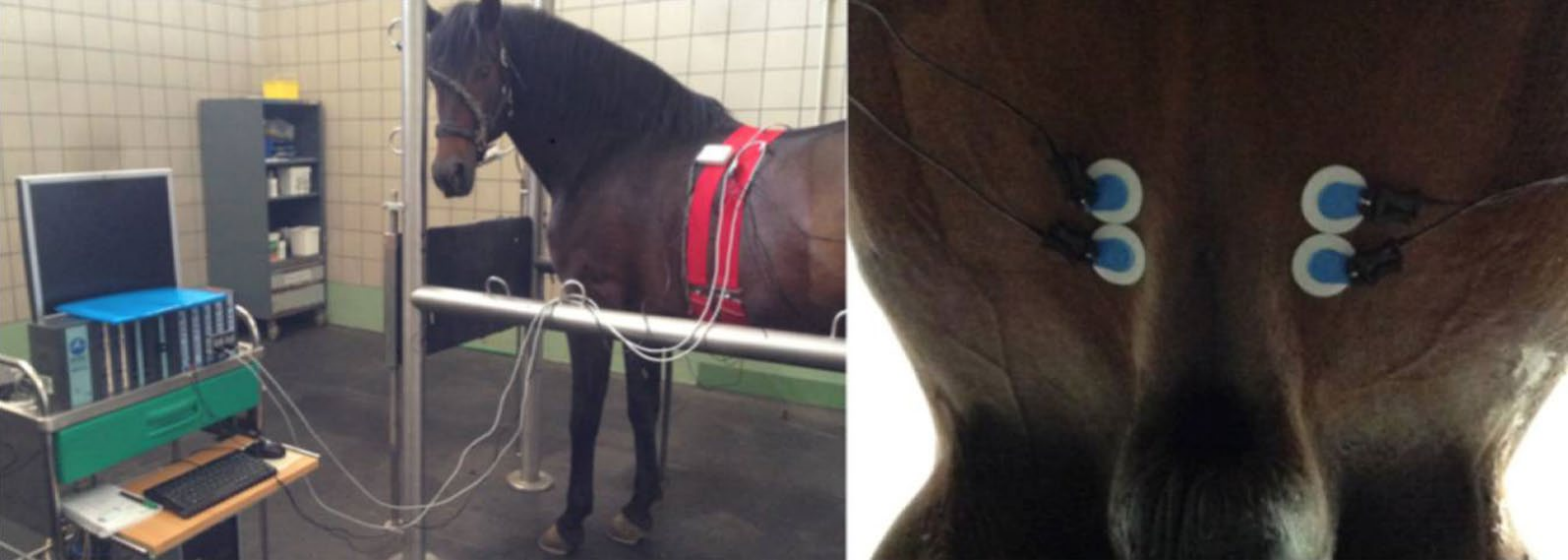
Electrodes on *Rectus abdominis* muscle

**Fig. 34:**
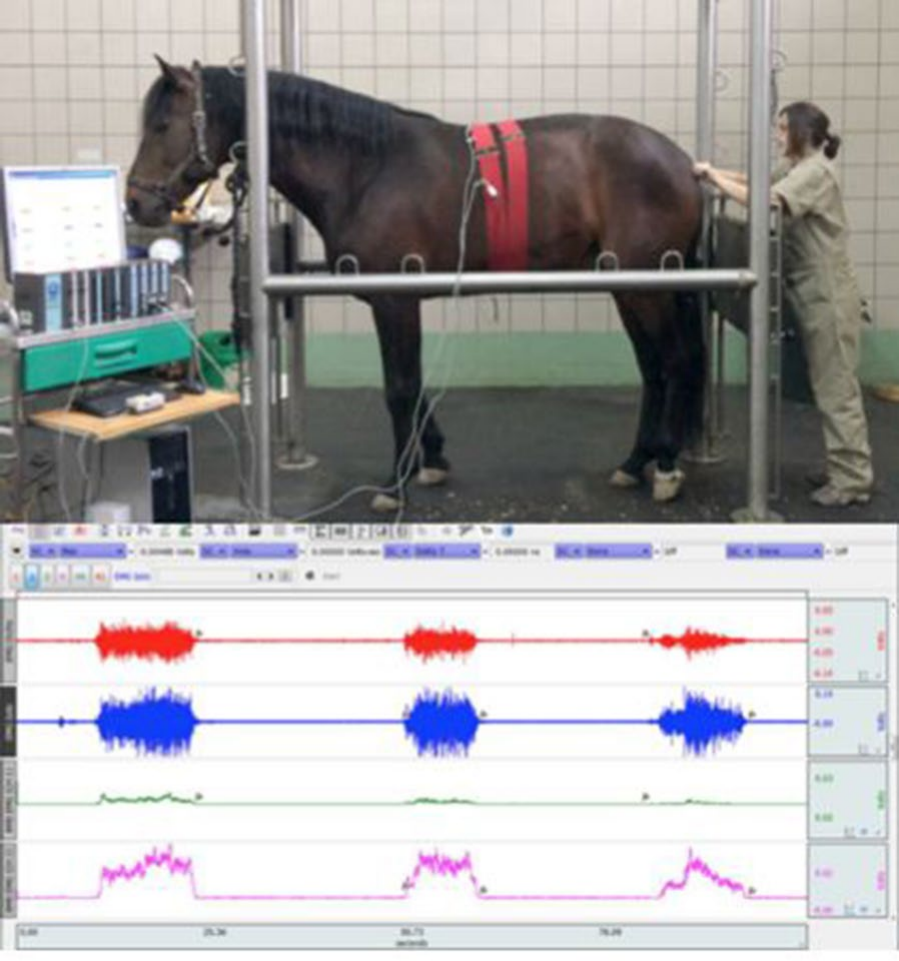
Manual stimulation and EMG recording

**Fig. 35:**
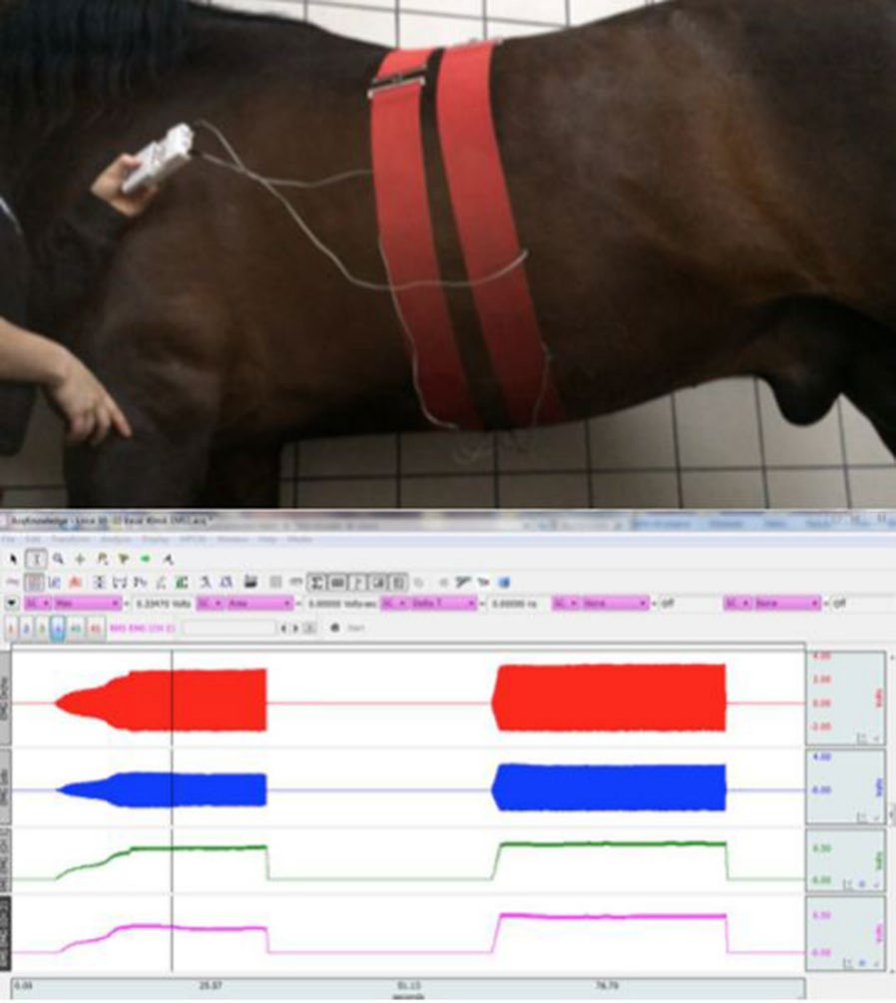
Electrical muscle stimulation and EMG recording

**Materials and methods:** Contraction of the *Rectus abdominis muscles* was induced with three different protocols in five horses and EMG was recorded (Fig. 33). Test A: three consecutive contractions induced by manual stimulation of the sacral area (Fig. 34). Test B: a 20 s contraction induced by electrical muscle stimulation (Fig. 35). Test C: 10 P-waves electrically induced. Root Median Square was obtained from test A and B, peak to peak from test C and Median frequency from test A.

**Results:** The uniformity of the EMG recording for tests B and C provided a more consistent measure of muscle force. All tests were useful for measuring muscle fatigue.

**Conclusions:** The described functional tests were useful to measure changes on muscle characteristics. These results open the possibility of using EMG as an assessing tool for therapeutic exercise.

**References**

1. Paulekas R, Haussler K. Principles and practice of therapeutic exercise for horses. J Equine Vet Sci. 2009; 29:870–93.

2. Stubbs NC, Kaiser LJ, Hauptman J, Clayton HM. Dynamic mobilisation exercises increase cross sectional area of musculus multifidus. Equine Vet J. 2011;43:522–29.

3. Tabor G. The effect of dynamic mobilisation exercises on the equine multifidus muscle and thoracic profile. Plymouth: University of Plymouth. 2015.

4. Gondin J, Guette M, Ballay Y, Martin A. Electromyostimulation training effects on neural drive and muscle architecture. Med Sci Sports Exerc. 2005; 37:1291–9.

## A29 The epaxial muscle cross-sectional area and fat content in Dachshunds with intervertebral disc herniation

### Anna F. Boström, Lotta Savolainen, Anu K. Lappalainen, Outi Laitinen-Vapaavuori

#### Small Animal Surgery, Department of Equine and Small Animal Medicine Faculty of Veterinary Medicine, University of Helsinki, Helsinki, Finland

##### **Correspondence:** Anna F. Boström - anna.bostrom@helsinki.fi

*Acta Veterinaria Scandinavica* 2016, **58(Suppl 2)**:A29

**Background:** The Dachshunds commonly suffer from intervertebral disc herniation (IVDH), which causes pain and neurological deficits [1]. In humans, IVDH is shown to cause back muscle atrophy where cross-sectional area (CSA) decreases and fat infiltration increases [2, 3]. Previous veterinary studies suggested similar findings when Dachshunds with IVDH were compared to dogs suffering from fibrocartilaginous embolism [4]. So far, no comparisons with healthy Dachshunds have been made.

**Objectives:** The objective was to compare the epaxial muscle CSA and fat content between healthy and IVDH Dachshunds.

**Materials and methods:** The magnetic resonance images of 10 healthy and 14 Dachshunds with thoracolumbar IVDH were evaluated retrospectively. One assessor used Osirix to analyze the CSA and fat content of the Multifidus and Longissimus dorsi muscles at the T12–L1 spinal segments. The assessor was blinded to the side, segment and background data of the dogs. The intra-rater reliability was tested with intra-class correlation coefficient. The difference between groups was analyzed with independent samples t test and Mann–Whitney U test. SPSS IBM statistics, version 23 was used, with level of significance set at 0.05.

**Results:** The intra-rater reliability was excellent for all measurements (ICC 0.92–0.99). There was no difference in the Multifidus or Longissimus dorsi CSA between the two groups, but the fat content was greater in the IVDH Dachshunds than in controls (P < 0.0001) for the evaluated muscles.

**Conclusions:** The increased fat content and non-affected CSA in the IVDH group agree with previous research [4], suggesting that fat tissue increases in the back muscles of Dachshunds with thoracolumbar IVDH.

**References**

1. Brisson B. Intervertebral disc disease in dogs. Veterinary clinics of North America. Small Anim Pract. 2010; 40:829–58.

2. Hyun JK, Lee JY, Lee SJ, Jeon JY. Assymmetric atrophy of multifidus muscle in patients with unilateral lumbosacral radiculopathy. Spine. 2007; 32:E598–602.

3. Battié MC, Niemeläinen R, Gibbons LE, Dhillon S. Is level- and side-specific multifidus asymmetry a marker for lumbar disc pathology? Spine J. 2012; 10:932–9.

4 Boström A, Hielm-Björkman A, Chang Y-M, Weller R, Davies E. Comparison of cross-sectional area and fat infiltration in dogs with and without spinal cord compression. Res Vet Sci. 2014; 97:646–51.

## A30 Objective gait analysis in cats with mild gait disturbances due to osteoarthritis

### Sarah Stadig^1^, Linda Lundström^2^, Anna Bergh^3^

#### ^1^Department of Clinical Sciences, Swedish University of Agricultural Sciences, Uppsala, Sweden, ^2^Hälsinge Small Animal Clinic, Hudiksvall, Sweden, ^3^Department of Anatomy, Physiology and Biochemistry, Swedish University of Agricultural Sciences, Uppsala, Sweden

##### **Correspondence:** Sarah Stadig - sarah.stadig@slu.se

*Acta Veterinaria Scandinavica* 2016, **58(Suppl 2)**:A30

**Background:** Osteoarthritis (OA) is a common cause of chronic pain and physical dysfunction in cats. Feline OA frequently affects bilateral joints and lameness is not a common clinical sign. Performing a clinical orthopaedic examination that yields reliable results is a challenge and the need for objective examination technique in cats is a necessity.

**Objectives:** The objective of the study was to compare the pressure mat results from a healthy group of cats with a group of cats with OA.

**Materials and methods:** The cats were grouped as OA (19) or healthy (12) based on their medical history, the owners´ assessment and findings from the physical examination. Three of the OA cats had low grade lameness, 16 walked with a stiff gait. The joints that had findings on the physical examination were radiographed. Most cats had more than one joint affected on physical examination. Each cat was allowed to walk over the pressure mat (Walkway™ System High Resolution HRV4) until at least two valid trials were obtained. The procedure was videotaped.

**Results:** The symmetry ratio front/hind (mean ± standard deviation) was 1.28 ± 0.18 for OA cats and 1.28 ± 0.13 for healthy cats (P 0.95).

**Conclusions:** There was no significant difference in the symmetry ratio or the PVF between healthy and OA cats. There was, however, a difference in the VI (Table 5). The differing VI is probably caused by the affected cats putting the same amount of load on the affected limb, but for a longer time. The stance time for each individual limb was calculated and it was a significantly longer in OA cats (t test). Improved diagnostics for cats with low grade lameness, bilateral and pauciarticular disease is needed.

**Table 5:** Peak vertical force (PVF) and vertical impulse (VI) for OA and healthy cats

	OA (19 cats)	Healthy (12 cats)	p value
	Mean ± SD	Mean ± SD	
PVF (% body weight)			
VF	30.7 ± 1.7	31.0 ± 2.9	0.55
VB	24.6 ± 3.5	24.7 ± 2.7	0.82
HF	30.3 ± 1.6	31.4 ± 3.3	0.17
HB	24.0 ± 3.4	24.1 ± 2.2	0.85
VI (% body weight × sec)			
VF	11.9 ± 1.9	10.3 ± 1.0	0.007
VB	9.1 ± 1.6	7.6 ± 1.5	0.006
HF	11.5 ± 1.7	10.5 ± 1.4	0.045
HB	8.8 ± 1.2	7.7 ± 1.4	0.01

*SD* standard deviation

## A31 Comparison of clinical examination results and radiography to diagnose feline osteoarthritis

### Sarah Stadig^1^, Linda Lundström^2^, Charles Ley^1^, Anna Bergh^3^

#### ^1^Department of Clinical Sciences, Swedish University of Agricultural Sciences, Uppsala, Sweden, ^2^Hälsinge Small Animal Clinic, Hudiksvall, Sweden, ^3^Department of Anatomy, Physiology and Biochemistry, Swedish University of Agricultural Sciences, Uppsala, Sweden

##### **Correspondence:** Sarah Stadig - sarah.stadig@slu.se

*Acta Veterinaria Scandinavica* 2016, **58(Suppl 2)**:A31

**Background:** Diagnosing chronic pain and physical disability caused by osteoarthritis (OA) in cats is a challenge. Currently, the diagnosis is based on a combination of the cat´s medical history, information from the cat owner, clinical examination and radiography. However, the absence of radiographic signs indicating OA does not equal a joint free from pathological changes causing pain.

**Objectives:** The objective was to compare the results from clinical examination with the radiographic findings in a group of cats with OA.

**Materials and methods:** Nineteen cats were diagnosed as osteoarthritic based on their medical history, the owners´ information and findings from the clinical examination. The cats were sedated (medetomidine and butorphanol) and the joints that had pathological findings on the clinical examination were radiographed. The right elbow was radiographed as a reference.

**Results:** On the clinical examination 63 joints out of 228 had pathological findings (Table 6). Of the 63 joints, 32 (51%) had radiographic findings of OA.

**Table 6:** Findings from clinical examination and radiographic examination of 19 cats with OA

Cat no.	R shoulder	L shoulder	R elbow	L elbow	R carpal	L carpal	R hip	L hip	R stifle	L stifle	R hock	L hock
1										[X]		
2									[X]	[X]		
3			X				[X]	[X]	[.]	[.]		
4			X	[.]			[X]	[.]				
5							[.]	[X]	[.]	[.]		
6									[.]			
7	[.]	[.]	[.]	[.]			[X]	[X]	[X]	[X]		
8							[X]	[.]		[X]		
9			[X]	X	[.]							
10			[.]	[.]			[.]	[X]				
11	[.]	[.]	[X]	[X]			[.]	[.]				
12^a^							[.]	[.]				
13		[.]	X	[X]			[.]		[.]			
14							[X]	[X]				
15							[.]	[X]				
16										[X]		
17										[X]		
18	[.]	[.]	[.]	[.]		[X]	[.]	[.]				
19	[.]	[.]	[.]	[.]	[X]	[X]	[X]	[X]				

*[.]* finding on clinical examination; *X* radiographic finding; *R* right; *L* left

^a^Cat number 12 was not radiographed and was therefore excluded from the analysis

**Conclusions:** Only every second joint that had pathological findings on clinical examination had radiographic findings indicating OA. This discrepancy is either caused by findings not detected by radiography, or by the cats experiencing discomfort during the clinical examination. There is need for improved diagnostic tools that can easily be used in a clinical setting.

## A32 Sensitivity and specificity for three clinical metrology instruments in osteoarthritic cats

### Sarah Stadig^1^, Linda Lundström^2^, Charles Ley^1^, Anna Bergh^3^

#### ^1^Department of Clinical Sciences, Swedish University of Agricultural Sciences, Uppsala, Sweden, ^2^Hälsinge Small Animal Clinic, Hudiksvall, Sweden, ^3^Department of Anatomy, Physiology and Biochemistry, Swedish University of Agricultural Sciences, Uppsala, Sweden

##### **Correspondence:** Sarah Stadig

*Acta Veterinaria Scandinavica* 2016, **58(Suppl 2)**:A32

**Background:** Osteoarthritis (OA) is a common cause of chronic pain and physical dysfunction in cats. Feline OA frequently affects bilateral joints and lameness is not a common clinical sign. Currently, the diagnosis is based on a combination of the cat’s medical history, client information, physical examination and radiography. Using client-based Clinical Metrology Instruments (CMI) in the form of questionnaires regarding changes in the cat´s daily behaviour and capabilities are important tools in identifying chronic pain and dysfunction due to OA. The validity of the CMIs needs to be tested in order to provide reliable tools in a clinical setting.

**Objectives:** The objective of the study was to test the sensitivity, specificity and discriminatory ability of three client-based CMIs in cats with OA and in healthy cats.

**Materials and methods:** The cats were grouped as OA (19) or healthy (12) based on their medical history, the clients´ assessment and findings from the physical examination. The joints that had findings on the physical examination were radiographed. Three CMIs designed for client-based use in cats with degenerative joint disease such as OA were tested: CMI I [1], CMI II [2] and CMI III [3]. CMI I and II contain questions where the client rates the cat´s ability, whereas CMI III contain binary questions (yes/no). A receiver operating characteristic (ROC) curve was drawn to calculate the Area Under the Curve (AUC) (Table 7).

**Table 7:** Sensitivity, specificity and AUC for the three CMIs

CMI	Sensitivity %	Specificity %	AUC
CMI I	59	83	0.82
CMI II	89	82	0.83
CMI III	95	56	0.86

CMI I had the highest specificity (83%), CMI III had the highest sensitivity (95%) and AUC (0.86), but the lowest specificity (56%). CMI II had the most evenly balanced results on all three analysed parameters

**References**

1. Benito J, Hansen B, Depuy V, Davidson GS, Thomson A, Simpson W, et al. Feline musculoskeletal pain index: responsiveness and testing of criterion validity. J Vet Intern Med. 2013; 27(3):474–82.

2. Zamprogno H, Hansen BD, Bondell HD, Sumrell AT, Simpson W, Robertson ID, et al. Item generation and design testing of a questionnaire to assess degenerative joint disease-associated pain in cats. Am J Vet Res. 2010; 71(12):1417–24.

3. Stadig S, Bergh A. The Feline Physical Function formula. 2011. Not published data.

## A33 Behaviour and respiratory rate after administration of methadone and methadone in combination with detomidine in horses

### Lena Olsén, Carina Ingvast-Larsson

#### Division of Pharmacology and Toxicology, Department of Biomedical Sciences and Veterinary Public Health, Swedish University of Agricultural Sciences, Uppsala, Sweden

##### **Correspondence:** Lena Olsén - Lena.Olsen@slu.se

*Acta Veterinaria Scandinavica* 2016, **58(Suppl 2)**:A33

**Background:** In treatment of nociceptive pain with opioids the full μ-agonists have highest efficacy. Methadone is a synthetic full μ-agonist and also an *N*-methyl-d-aspartate (NMDA) receptor antagonist used in horses. Opioids might also cause excitement in the horse and respiratory depression especially when used together with other drugs affecting the respiration. The α_2_-agonists are used in horses for sedation and analgesia both separately and in combination with opioids. A combination of methadone and the α_2_-agonist detomidine may be useful in pain management but may also cause effects on behaviour and respiratory rate.

**Objectives:** The aims were to investigate the effects on behavior and respiratory rate after treatment with methadone IV or methadone IV in combination with detomidine IM in order to evaluate and possibly diminish adverse effects in the treatment of horses.

**Materials and methods:** The study was randomized, blinded and placebo controlled with cross-over design. Eight Standardbred horses were treated with (1) methadone IV (0.2 mg/kg) in a total volume of 20 mL over 5 min together with placebo (saline) IM or (2) 20 mL methadone IV (0.1 mg/kg) over 5 min together with detomidine IM (0.01 mg/kg) or (3) equivalent volumes saline IV and IM. The effects on behavior and respiratory rate were examined.

**Results:** After methadone administration the horses displayed behavioral changes such as staggering for a short period, head tremors and looking vigilantly around. The behaviors licking, nodding head, picking hay, tail flapping, skin twitching and scraping with front leg were more frequent after treatment with methadone compared to the control. There were no differences in respiratory rate between methadone and saline treatments.

After methadone/detomidine administration the horses showed drowsiness, snored, stood with dropped head or head supported by the wall or the crib during the first one to three hours. In addition, a few horses were sweating. The respiratory rate was lowered between one and three hours after administration of the drug combination compared with placebo (*P* = 0.02).

**Conclusions:** When the horses were treated with methadone they showed drug-related agitation which has been described in horses treated with different opioids. The combination of methadone/detomidine seems to prevent excitement caused by opioid exposure. A lowered respiratory rate is also a known non-wanted effect of opioids but in this study no such adverse effect was observed after solely methadone administration but the combination methadone/detomidine induced decreased respiratory rate.

**Acknowledgements:** Supported by The Swedish-Norwegian Foundation for Equine Research.

## A34 Pain assessment in the horse: effect of methadone alone and methadone in combination with detomidine in horses

### Lena Olsén, Carina Ingvast-Larsson

#### Division of Pharmacology and Toxicology, Department of Biomedical Sciences and Veterinary Public Health, Swedish University of Agricultural Sciences, Uppsala, Sweden

##### **Correspondence:** Lena Olsén - Lena.Olsen@slu.se

*Acta Veterinaria Scandinavica* 2016, **58(Suppl 2)**:A34

**Background:** In pain management it is important to assess pain and pain relief in a robust way. Thermal threshold are experimentally used in pain assessment for several species including horses. Opioids such as methadone are effective in treatment of nociceptive pain but may also cause effects on traits used for assessing pain such as cortisol levels. Also alpha2-adrenoceptor agonists (α_2_-agonists) such as detomidine are commonly used for analgesia and sedation both separately and in combination with opioids. The possible influence of detomidine of the effects of methadone is not well studied in horses.

**Objectives:** The aims of this study were to reveal possible drug effects on pain assessment tools and to optimize pain treatment in horses. The effects on plasma cortisol levels was examined and the analgesic effect was measured with a thermal threshold testing system adopted for horses was used after treatment with methadone IV or methadone IV in combination with detomidine IM.

**Materials and methods:** The study was a randomized blinded placebo controlled with *cross over* design. Eight Standardbred horses were treated with (1) methadone IV (0.2 mg/kg) in a total volume of 20 mL over 5 min together with placebo (saline) IM or (2) 20 mL methadone IV (0.1 mg/kg) over 5 min together with detomidine IM (0.01 mg/kg) or (3) equivalent volumes placebo (saline) IV and IM. The cortisol levels and analgesia after administration of methadone alone and together with detomidine were examined. Blood samples were collected and the plasma concentrations of cortisol were quantified with a commercial cortisol ELISA kit validated in horses.

**Results:** After both administration of methadone and the combination methadone/detomidine a rise in the plasma cortisol concentration compared to control (*P* < 0.05) occurred. The thermal threshold was elevated and analgesia was apparent for 1 h when treated with methadone compared to saline (*P* = 0.001) and for up to 2 h when combining methadone and detomidine (*P* = 0.02).

**Conclusions:** Both methadone and the combination methadone/detomidine induced the release of plasma cortisol in horses *per se*. Plasma cortisol concentration is not a useful tool to assess stress or pain in horses treated with methadone. The duration of the analgesic effect of methadone were short but when combined with detomidine the duration was prolonged. For extended analgesia a constant rate infusion of methadone could be used.

**Acknowledgements:** Supported by The Swedish-Norwegian Foundation for Equine Research.

## A35 Optimizing thermal testing system in the horse

### Lena Olsén, Carina Ingvast-Larsson

#### Division of Pharmacology and Toxicology, Department of Biomedical Sciences and Veterinary Public Health, Swedish University of Agricultural Sciences, Uppsala, Sweden

##### **Correspondence:** Lena Olsén - Lena.Olsen@slu.se

*Acta Veterinaria Scandinavica* 2016, **58(Suppl 2)**:A35

**Background:** Accurate, repeatable and reliable pain recognition and quantification of analgesia are essential for development of effective analgesic protocols and necessary for adequate pain management. Thermal threshold testing techniques have valuable roles in both the identification of altered nociceptive function and the pre-clinical evaluation of analgesics in horses. When using test systems, definition of a clear cut end-point of stimulation, such as skin twitching, shaking or hoof withdrawal is crucial for reliable and repeatable determination of the nociceptive threshold. End-point behaviour might be reflex related or may represent conscious perception of pain but could also be influenced by the environment and the horse’s willingness to show pain.

**Objectives:** The overall aim was to optimise the reliability and repeatability of the testing procedures in a future study assessing analgesic treatment protocols. The optimal settings of the testing system was evaluated. Furthermore, the type of end-point behavior showed by different horses was investigated.

**Materials and methods:** Fifteen Standardbred horses were evaluated using a thermal threshold testing system adopted for use in horses (Topcat Metrology). All horses were tested by heating with a thermal probe on skin at the withers until an end-point behaviour (threshold temperature) or a cut-out temperature (58 °C) was reached. The type of end-point behaviour, the willingness to wear the testing system and to show pain related behaviour was evaluated. Also the skin temperature before heating, the rate of heating (0.5 vs 1.0 °C/s) and repeatability was examined. The study was blinded with cross-over design.

**Results:** No differences in mean threshold temperature (49.5 and 50.7 °C, respectively) between the rates of heating of 0.5 vs 1.0 °C/s, nor in delta temperature (14.0 and 14.4 °C, respectively). There were individual differences in type of behaviour and easiness to observe their end-point behaviour and some horses did not produce an identifiable end-point behaviour before cut-out temperature. We identified and selected horses showing easy recognized skin twitch as end-point behavior to be included in our future study of analgesic treatments.

**Conclusions:** Thermal threshold testing appears not to be an appropriate test technique in all individual horses. As higher cut-out temperatures could possibly damage the skin it is not appropriate to include more “stoic” horses. This implies that if pain is more easily identified in extrovert individuals the relationships between pain behavior and personality in horses should be clarified for better study design in pain management.

**Acknowledgements:** Supported by The Swedish-Norwegian Foundation for Equine Research.

## A36 Can physical therapy improve the sleep-wake rhythm of older dogs?

### Renata Diniz, Cristina Nicolau, Antonio Gamundi, Mourad Akaarir

#### Department of Biology, University of the Balearic Islands, Mallorca, Spain

##### **Correspondence:** Renata Diniz - renatadiniz23@gmail.com

*Acta Veterinaria Scandinavica* 2016, **58(Suppl 2)**:A36

**Background:** A number of studies with human patients support the importance of a robust day-night sleep rhythm in order to remain healthy. With the elderly, some symptoms such as alterations of the sleep, sensory and motor deficits can be related with disorders of the circadian rhythm. Few researches about dog’s circadian system have been made, likely because it is difficult to evaluate under normal living conditions.

**Objectives:** To compare the sleep-wake rhythm of older dogs pre and post physical rehabilitation.

**Materials and methods:** The study was performed with seven healthy dogs (10–14 years old, 21.8 ± 8.9 kg) of different breeds. All dogs were examined and evaluated by physical rehabilitation. All dogs had characteristic symptoms of aging: poor mobility and a few points of chronic pain, but they did not have any significant pathology, nor took prescription medications. After the treatment (4–6 weeks) they were evaluated again to compare before and after the physical therapy. The life condition was the same during all treatment, like food, water or specific environment. The rehabilitation was individual and included physical therapy and kinesiotherapy, according to the need of each dog, twice a week. The circadian rhythm was measured per 3 days (day and night) by actimetry using a sensor of rest-activity (Hobo, Massachusetts, USA) and another of temperature (iButton Viewer 32). The sensors were positioned on the dog’s collar. All dogs had a record card with historical and evaluation of pain and clinic status. The rhythm of temperature and activity were analyzed with the software “Circandianware” v7.1.1 and “Actiwatch” 2001, which allows both the parametric analysis and nonparametric analysis.

**Results:** All seven dogs had better physical condition after the treatment. The circadian parameters like the sleep or awake time, sleep efficiency, fragmentation sleep index and acrophase, were all very similar pre and post treatment. On the other hand, the degree of regularity in the activity-rest pattern (interdaily stability) of the 24 h rhythm was increased after the rehabilitation. In addition, the Rayleigh test (a statistic test used to determine the pattern of a particular rhythm) was higher post rehabilitation and indicates the homogeneity of the daily phases. These results have a direct relationship with quality of life and could indicate a better sleep-wake rhythm after the treatment.

**Conclusions:** The present study demonstrates that physical rehabilitation improves the sleep-wake rhythm in older dogs, suggesting a good way to maintain their quality of life.

## A37 Evaluation of the reliability of real-time ultrasonography to measure muscle thickness of the canine middle gluteal muscle

### Elizabeth Roberts, Leander McLennan, Helen C. Cartildge, Lucy K. M. Evans, Stephen Baugh

#### Animal Production, Welfare and Veterinary Sciences Department, Harper Adams University, Edgmond, Shropshire, TF10 8NB, UK

##### **Correspondence:** Elizabeth Roberts - broberts@harper-adams.ac.uk

*Acta Veterinaria Scandinavica* 2016, **58(Suppl 2)**:A37

**Background:** Development of physiotherapy techniques and rehabilitation protocols is dependent on accurate measurements of outcome parameters [1]. Physiotherapists frequently focus on helping build muscle strength, yet few objective tests to evaluate the effectiveness of these treatments exists [1–3]. Maximum isometric force of a muscle correlates with its cross sectional area (CSA) [4]. Therefore, in situations where a muscle cannot be contracted voluntarily or strength measurements are inappropriate, changes in muscle size, [measured in CSA or thickness (MT)], are used as an indication of muscle strength [4, 5].

**Objectives:** The study was an operator-blind clinical trial of repeated ultrasonography (ULT) to determine a standardised method for MT measurement in the middle gluteal muscle of canines in the clinical setting.

**Materials and methods:** ULT measurements were taken from ten healthy canines, by three operators (operator one inexperienced, operator two moderate experience, and operator three experienced), each following a protocol that evaluated three repeated measurements of both hind limbs to evaluate MT of cross-sectional (CS) and longitudinal (LT) views. Measurements were taken one-third of the distance between the origin on the wing of the ilium and the insertion onto the greater trochanter of the femur.

**Results:** Good intra-rater reliability was found with MT measurements of both CS and LT views, with the operator variability for the right leg ranging from 0.09 to 0.23 cm^2^, and the left 0.27 to 0.41 cm^2^. There was significant difference between limbs using ANOVA, the left limb was considered not clinically reliable for all operators due to variability values being twice that of the right limb. No significant differences between the readings for the operators, determined that there was good inter-rater reliability for CS (P = 0.55, *cv* = 63.7%) and LT (P = 0.298, *cv* = 61.3%) measurements, tested using ANOVA one-way correlation coefficient testing. Table 8 shows between operator correlations, comparing these with Pearson’s critical value of 0.6319 at P = 0.05 indicates: strong correlations between operators two and three, and moderate correlations between one and two, and one and three.

**Table 8:** Correlation coefficients for operator variability

Operators	Left CS	Right CS	Left LT	Right LT
1 v 2	0.71	0.50	0.83	0.69
2 v 3	0.82	0.75	0.84	0.80
1 v 3	0.62	0.71	0.68	0.65

**Conclusions:** This study indicates that ULT for both CS and LT measurements appears to be a reliable tool for measuring MT in vivo in canines. The absence of scientifically proven and quantified measurements of MT means that no conclusions regarding the accuracy of measurements can be made. Further research is required to demonstrate that ULT is measuring the actual MT, to test the reliability of the CS verse LT measurements, and determine the reasoning behind the left limb results.

**References**

1. Millis DL, Scroggs L, and Levine D. Variables affecting thigh circumference measurements in dogs. In: Proceedings of the First International Symposium on Rehabilitation and Physical therapy in Veterinary Medicine (IAVRPT) 1999; Corvalis.

2. Stokes M, Young A. Measurement of quadriceps cross sectional area by ultrasonography: a description of the technique and its application to physiotherapy. Physiother Prac. 1986; 2:31–6.

3. Reeves ND, Maganaris CN, and Narici MV. Ultrasonography assessment of human skeletal size. Eur J Appl Physiol. 2004; 91:116–8.

4. Bruce SA, Phillips SK, Woledge RC. Interpreting the relation between force and cross sectional area in human muscle. Med Sci Sports Exerc. 1997; 29(5):677–83.

5. Fukunga T, Miyatani M, Tachi M, Kouzaki M, Kawakami Y, Kaneshi H. Muscle volume is a major determinant of joint torque in humans. Acta Physiol Scand. 2001; 172(4):244–55.

## A38 The effect of an elastic resistance band around the hindquarters on equine dorsoventral back kinematics

### Pernilla Stenfeldt^1,2^, Cajsa Ericson^1,3^, Inger Jacobson^4^

#### ^1^Veterinary Physiotherapy, University of Liverpool, Leahurst, Liverpool, CH64 7TE, UK, ^2^Hästrehab & Byggkonsult I Ängelholm AB, Vanstadsvägen 121, 262 91, Ängelholm, Sweden, ^3^Animotion Rehab, Ankdammsgatan 18, 171 43, Solna, Sweden, ^4^Division of Health Sciences, Luleå University of Technology, 971 87, Luleå, Sweden

##### **Correspondence:** Pernilla Stenfeldt - pernilla.stenfeldt@telia.com

*Acta Veterinaria Scandinavica* 2016, **58(Suppl 2)**:A38

**Background:** In horses, lameness and back problems caused by overloading and repetitive stress due to poor balance and working posture are common. The Equiband™ system is suggested to improve dynamic stability and posture by providing proprioceptive stimulation to the hindquarters.

**Objectives:** To investigate the effect of the Equiband™ resistance band around the hindquarter on equine thoracolumbar dorsoventral back kinematics.

**Materials and methods:** A paired controlled intervention study with seven privately owned horses, considered sound by their owners and in regular work were included in this study, lasting over 3 days. Thoracolumbar dorsoventral kinematics were measured at walk and trot in-hand on a straight line under 3 conditions: (1) with the Equiband™ hindquarter band attached to a single girth. (2) With only the single girth and (3) Control (no girth or band). Reflective markers were attached along the back and 20 high speed motion cameras and their associated data management software captured and processed the data. The mean motion differences for segments L5, L3, T15 and T12 were analysed using a student’s t test on normally distributed data and Wilcoxon signed ranks test on non-normally distributed data.

**Results:** Significant differences were detected in both walk and trot for ROM and separate flexion/extension values for all three conditions. A stabilizing effect with the Equiband™ was seen in the lumbar area and at T12, except for L5 in walk where an increased ROM was detected compared to the control condition. At T15, an increased extension occurred with the Equiband™. The girth showed a significant effect especially at T12 and T15 similar to the Equiband™ effect.

**Conclusions:** The Equiband™ hindquarter band is a powerful tool for altering the thoracolumbar back kinematics. Stabilisation of the lumbar region may confer improved working posture and an increased core stability. Further studies are needed to investigate the additional effect of the abdominal elastic band.

## A39 Assessment and reassessment of movements in dogs at a treadmill walk following cranial cruciate ligament surgery—the use of kinematic variables in a rehabilitation perspective

### Pia Gustås^1^, Linnéa Söderberg^1^, Lennart Sjöström^2^, Robert Colborne^3^, Anna Byström^4^

#### ^1^Department of Clinical Sciences, Faculty of Veterinary Medicine and Animal Husbandry, Swedish University of Agricultural Sciences, Box 7054, 750 07, Uppsala, Sweden, ^2^Evidensia Strömsholm Referral Animal Hospital, Djursjukhusvägen 11, 734 94, Strömsholm, Sweden, ^3^Institute of Veterinary, Animal and Biomedical Sciences, Massey University, Private Bag 11-222, Palmerston North, 4442, New Zealand, ^4^Department of Anatomy, Physiology and Biochemistry, Faculty of Veterinary Medicine and Animal Husbandry, Swedish University of Agricultural Sciences, Box 7011, 750 07, Uppsala, Sweden

##### **Correspondence:** Pia Gustås - pia.gustas@slu.se

*Acta Veterinaria Scandinavica* 2016, **58(Suppl 2)**:A39

**Background:** The general objective of gait analysis is to identify deviations from normal gait patterns. Kinematic studies have identified differences in limb movement patterns between normal and distinctly lame dogs. Reassessment of gait at multiple time points during rehabilitation after surgical interventions, like TPLO or other cranial cruciate ligament surgery depends on accurate identification of limb landmarks for consistency of kinematic marker placement, and consistency of testing parameters, like walking/trotting velocity. Variability is inherent in gait, and changes occurring as a result of treatment must be greater than normal, within-session variability to be detected.

**Objectives:** To investigate if basic gait variables can be used for detection of gait changes in dogs following cranial cruciate ligament surgery at two separate occasions during recovery >2 months after surgery.

**Materials and methods:** Six dogs were recorded at walk on a treadmill at two different sessions one month apart. The first session was more than 2 months after surgery for unilateral cranial cruciate ligament rupture. Reflective markers were placed on palpable landmarks on all four limbs and along the back. Fourteen Qualisys infrared light emitting video cameras registered marker positional data at 500 Hz. Tracking was done in Qualisys Track Manager, while stride split into stance and swing phases and further calculations were done in a customized Matlab script. Variables used were stride time, stance time and relative stance time (duty factor); the sagittal angular displacement of carpal, elbow, tarsal and stifle joints, and the pro- and retraction angle of fore and hind limbs. Mean values for each variable per dog, per session, were calculated and statistically analysed with ANOVA, the mixed procedure.

**Results:** Stifle joint angular displacement, for both surgically treated and contralateral hind limbs, increased (P < 0.1) between the first and second sessions. For the ipsi-lateral carpus, joint angular displacement was larger (P < 0.1) at the first session than the second. The protraction-retraction, i.e. the whole limb pendulum, was smaller in the surgical hind limb at both sessions. Stride time was longer in the second session. Stance time didn’t differ either between limbs or sessions. However, relative stance time (duty factor) was shorter (P < 0.1) in the ipsilateral compared to the contralateral hind limb in both sessions.

**Conclusions:** This pilot study showed that kinematic analysis can identify small differences through the late recovery period following cranial cruciate ligament surgery, although a full study with a larger sample size and kinematic variables needs to be performed.

## A40 The effect of kinesiotape on range of motion in flexion–extension of the thoracolumbar back in the trotting horse

### Cajsa Ericson^1,2^, Pernilla Stenfeldt^1,3^, Inger Jacobson^4^

#### ^1^Veterinary Physiotherapy, University of Liverpool, Liverpool, UK, ^2^Animotion Rehab, Ankdammsgatan 18, 171 43, Solna, Sweden, ^3^Hästrehab & Byggkonsult I Ängelholm AB, Vanstadsv 121, 262 91, Ängelholm, Sweden, ^4^Division of Health Sciences, Luleå University of Technology, 971 87, Luleå, Sweden

##### **Correspondence:** Cajsa Ericson - cajsaericson@gmail.com

*Acta Veterinaria Scandinavica* 2016, **58(Suppl 2)**:A40

**Background:** Kinesiotape in horses is a commonly used method in equine physiotherapy and veterinary rehabilitation. The method theoretically stimulates mechanoreceptive and proprioceptive sensory pathways from the taped region that in turn modulates the neuromuscular activity and locomotor function so alteration of activation, locomotion and/or range of motion (ROM) can be achieved.

**Objectives:** The aim of this study was to determine if kinesiotape applied to the abdominal muscles would affect the ROM in flexion–extension in the thoracolumbar back of the trotting horse.

**Materials and methods:** A paired experimental study, convenience sample with each horse as its own control. Eight horses, 5–15 years (mean age 8.75 years) were included.

The horses were trotted up in hand on a straight line for 2 × 50 m with and without kinesiotape in a randomized order. The mean speed was 3.2 m/s. An objective analysis of the ROM in flexion–extension of the thoracolumbar back was performed using 20 infrared motion capture cameras. Data was collected from 16 reflective skin surface markers placed on the levels of T6, T12, T15, L3, L5, tuber sacrale and S3 and was converted into 3D data. Flexion–extension ROM was measured, tabulated and tested for differences between intervention and not intervention state. Paired t tests, normality tests and 1-Sample Wilcoxon test were used to assess the effects of the kinesiotape.

**Results:** No significant (P < 0.05) changes in ROM in flexion–extension of the thoracolumbar back in trot were shown in this group of horses. Individual variation, but within individual trends only, some changes were shown indicating individual strategies to manoeuvre the stimuli from the kinesiotape, but the changes were too small for statistical significance.

**Conclusions:** This study did not show any significant effect of kinesiotape in ROM in extension-flexion of the back. More research in this popular and clinically used method is needed to fully understand the mechanisms—in both human and horses.

## A41 Case report: bilateral superficial digital flexor tendon disruption with flexor carpi ulnaris tendinopathy in the forelimbs of a dog

### Marti Drum^1^, Marie de Swarte^2^, Federica Morandi^2^, José Guevara^1^, Darryl Millis^1^

#### ^1^Department of Small Animal Clinical Sciences, Canine Arthritis, Rehabilitation, Exercise and Sports Medicine (CARES) Section, University of Tennessee College of Veterinary Medicine, Knoxville, TN, USA, ^2^Department of Small Animal Clinical Sciences, Radiology Section, University of Tennessee, TN, USA

##### **Correspondence:** Marti Drum - mdrum@utk.edu

*Acta Veterinaria Scandinavica* 2016, **58(Suppl 2)**:A41

**Background:** Superficial Digital Flexor (SDF) tendinopathies of the forelimbs are very common in horses, but have not been reported in dogs to date. SDF luxation of the rearlimbs is well-documented in dogs, but occurs due to tearing of the tendinous retinaculum, not the SDF tendon body or insertion. This is a case of a bilateral SDF tendinopathy with complete fiber disruption and intact, abnormal flexor carpi ulnaris tendons.

**Fig. 36:**
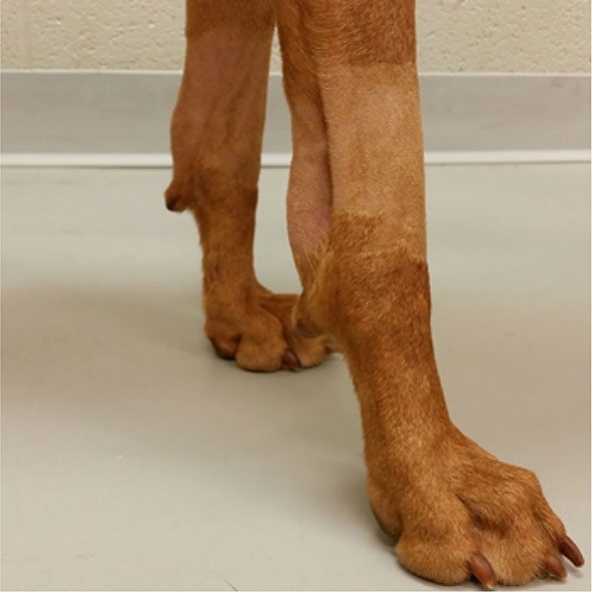
Bilateral thickening of superficial digital flexor and flexor carpi ulnaris tendons

**Fig. 37:**
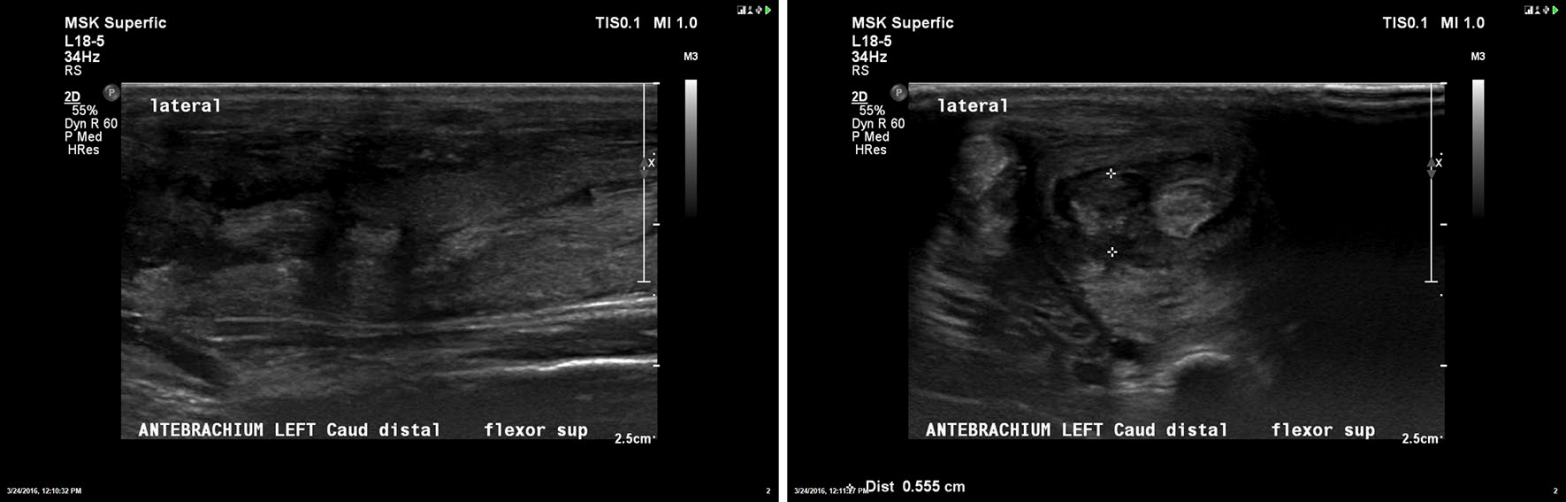
Transverse and longitudinal images of the left superficial digital flexor tendon at the level of the distal antebrachium

**Fig. 38:**
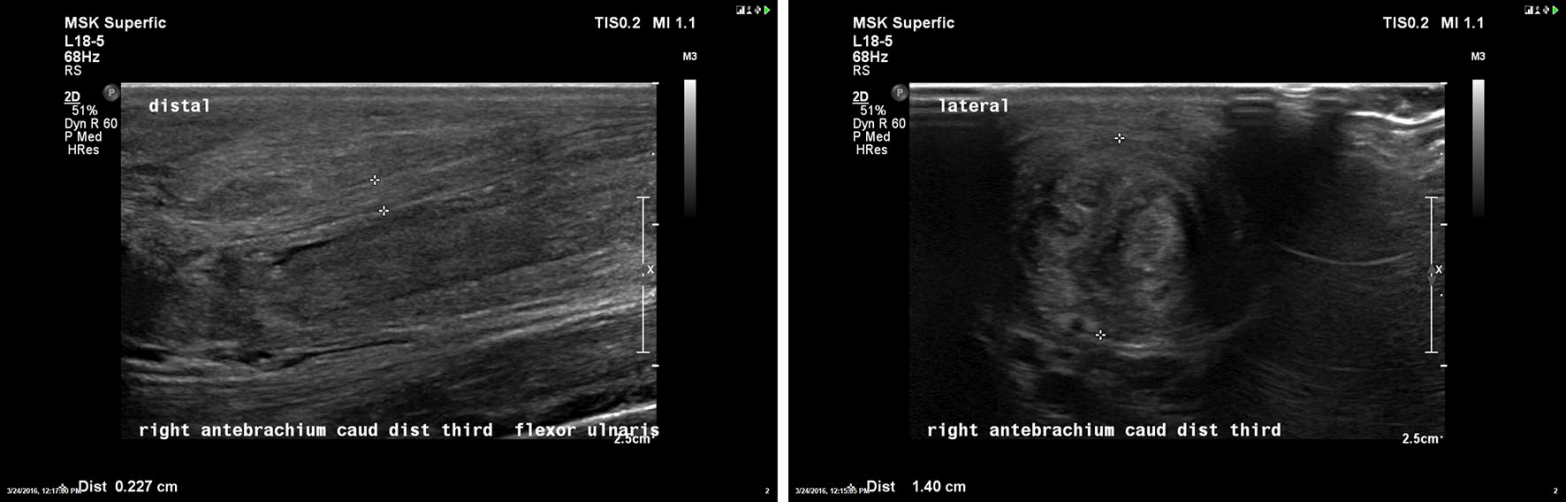
Transverse and longitudinal images of the right superficial digital flexor tendon at the level of the distal antebrachium

**Case description:** A 6 year-old intact male Vizsla presented for an intermittent right forelimb lameness of approximately 3 months duration that had partially responded to rest and anti-inflammatory therapy. An intermittent left forelimb lameness also developed 6 weeks prior to presentation. No history of a traumatic event was noted. Obvious left forelimb lameness, bilateral swelling and deformity of the caudodistal antebrachium and palmar carpus were noted at presentation (Fig. 36). Pain was elicited on palpation of the firm swelling bilaterally but carpal hyperextension was not present. A severe, bilateral lameness was present on force platform gait analysis with the right forelimb being significantly worse than the left. Tendons of both the SDF and flexor carpi ulnaris appeared thickened with a disorganized fiber pattern on ultrasound evaluation, but complete disruption of only the SDF tendon was noted bilaterally in the distal antebrachium (Figs. 37, 38). Ultrasound guided intralesional injection of Platelet Rich Plasma (PrP) and Extracorporeal Shockwave Therapy (ESWT) were administered at presentation and repeated again 2 weeks later. ESWT was also repeated in the right forelimb only following recheck ultrasound at 6 weeks after initial diagnosis. Activity was limited to leash walks for the first 2 weeks after initial treatment with gradual return to light activity by 6 weeks post treatment. Repeat force plate evaluation at 2 weeks after initial treatment showed dramatic (25%) improvement in weight bearing (PVF) bilaterally, and near normal weight bearing was present 6 weeks post-initial treatment with no visible lameness. However, a mild right forelimb lameness was still noted at 6 weeks (6.6% difference) with minimal to no tendon healing on repeat ultrasound exam.

**Discussion:** This unique case of forelimb SDF tendinopathy is intriguing without the presence of obvious trauma, laceration, carpal hyperextension, or tearing of flexor carpi ulnaris tendons. Clinical response to treatment was greater than expected and slow ultrasonographic healing was anticipated. Follow-up is ongoing. The combination of PrP and ESWT appeared more successful than rest and anti-inflammatories.

## A42 Conservative management of cranial cruciate ligament disease in dogs

### Darryl Millis, Dawn Hickey, Marti Drum

#### CARES Center, University of Tennessee College Of Veterinary Medicine, Knoxville, TN, USA

##### Correspondence: Darryl Millis - dmillis@utk.edu

*Acta Veterinaria Scandinavica* 2016, **58(Suppl 2)**:A42

**Background:** Surgical management of cranial cruciate ligament disease (CCLD) in dogs is usually recommended. Recently, owners have pursued conservative management of CCLD for a number of reasons. Information regarding conservative treatment and outcomes is necessary to design prospective studies to evaluate treatment efficacy.

**Objectives:** The main objective of this study was to review cases of CCLD managed conservatively to identify potential factors that may be useful in designing a prospective clinical study to evaluate the efficacy of nonsurgical treatment.

**Materials and methods:** The medical records of dogs undergoing conservative management of CCLD from January 2013 to March 2016 were evaluated. Information recorded included age, weight, breed, sex, clinical findings, medical treatment, rehabilitation treatment, and outcome if known. Data were evaluated for factors that may have a positive outcome with conservative management.

**Results:** Records of 25 dogs with conservative management of CCLD were evaluated. The mean age was 7.2 years (±2.9, range 3–13), with 15 spayed females, one intact female, nine neutered males, and one intact male dog. Professional rehabilitation was performed in 23, and home exercise programs in two dogs. A mean of 11.1 (±10.7, range 0–40) visits were made for rehabilitation, with 10.6 (±13.5, range 0–60) total weeks of treatment. Platelet rich-plasma injections were performed in 6 dogs. Five dogs required surgery because of unsatisfactory results of conservative management. Subjective outcome was very good (n = 4), improved (n = 9), poor/required surgery (n = 9), unknown (n = 1), or dogs died or had serious illness during treatment (n = 2). Other treatments included polysulfated glycosaminoglycans (10), NSAIDs or tramadol (20), nutraceuticals (19), omega-3 fatty acids (14), and weight loss (8). Although firm conclusions could not be determined from this record review, there was a tendency for dogs to have better outcomes with more intense treatments, including aquatic therapy, proprioceptive training, joint motion exercises, platelet-rich plasma injections, pharmaceuticals, and nutraceuticals. The greatest reasons for choosing conservative management of CCLD included advanced age of the dog, other major health concerns, uncertainty of surgery, possibility of surgical complications or aftercare management concerns.

**Conclusions:** More intensive management, including extensive rehabilitation, pharmaceutical management, and nutraceuticals may result in better outcomes with conservative management of CCLD. However, owners should be aware of the cost and length of treatments, and the risk of unsatisfactory outcomes.

## A43 Rehabilitation and outcomes of dogs with Fibrocartilaginous Embolic Myelopathy

### Dawn Hickey, Darryl Millis, Marti Drum

#### University of Tennessee College of Veterinary Medicine, Knoxville, TN, USA

##### Correspondence: Darryl Millis - dmillis@utk.edu

*Acta Veterinaria Scandinavica* 2016, **58(Suppl 2)**:A43

**Background:** Fibrocartilaginous embolic myelopathy (FCE) is a common neurologic condition. Patients are often presented for physical rehabilitation. Functional recovery reportedly depends on the severity of deficits. Knowledge of outcomes in dogs receiving rehabilitation would be helpful to veterinarians and owners.

**Objectives:** The objectives of this study were to review the neurologic findings of cases with FCE, treatments performed, and patient outcomes. We hypothesized that dogs receiving rehabilitation would have improved outcomes compared with historical outcomes.

**Materials and methods:** Records of patients with a diagnosis of FCE from September 2007 to March 2016 were reviewed. All dogs had an MRI performed. Neuroanatomic diagnosis, whether or not rehabilitation was performed, and if so, the forms of treatment, were recorded. Patient outcomes were recorded as normal, functional (walks without assistance with minimal gait abnormalities), improved, and not functional.

**Results:** Sixty-one dogs had MRI findings consistent with FCE. The mean age was 6.6 years and mean weight was 21.6 kg. Miniature schnauzers (11), mixed breeds (9), and Labrador retrievers (6) were overrepresented. Neuroanatomic localization included forelimb monoparesis (2), hemiparesis (8), pelvic limb monoparesis (5), pelvic limb paraparesis (17), pelvic limb paraplegia (16), tetraparesis (10), and tetraplegia (3). 29 dogs had professional rehabilitation (PR). With PR, one developed normal function, 17 functional recovery, eight improved, two were not functional, and one had functional recovery in one pelvic limb but not the other. Deep pain was initially absent in six dogs receiving PR; two became functional, one improved, two were nonfunctional, and one regained function in one pelvic limb but not the other. Fourteen dogs in the PR group had no motor function initially; nine regained function, three improved, one was not functional, and one regained function in one pelvic limb but not the other. Although adequate follow-up information was not available for the 32 dogs receiving no rehabilitation, 10 had no initial motor function and four had no deep pain.

**Conclusions:** Dogs with no deep pain or loss of motor function were 50% more likely to receive PR. Most dogs with PR had functional recovery (62%) or were improved (28%), despite 21% having no deep pain and 48% having no motor function at time of presentation. These results compare favorably to previous reports of recovery in patients with FCE receiving no PR.

## A44 Cardiovascular parameters of exercising pet dogs

### Ellen Camp, Rachel Dickson, Jose Guevara, Marti Drum, Darryl Millis

#### CARES Center, University of Tennessee College Of Veterinary Medicine, Knoxville, TN, USA

##### Correspondence: Darryl Millis - dmillis@utk.edu

*Acta Veterinaria Scandinavica* 2016, **58(Suppl 2)**:A44

**Background:** Information exists regarding cardiovascular changes with exercise of sled dogs and racing Greyhounds. However, there is little information regarding cardiovascular responses in exercising household pets. Additionally, there is little information regarding cardiovascular parameters for safe exercise recommendations without causing excess fatigue. Because cardiovascular parameters in pet dogs may differ from those of elite athlete dogs, we collected data from dogs not currently in a conditioning program to formulate recommendations regarding safe exercise levels.

**Objectives:** Our main objective was to measure cardiovascular, respiratory, and thermal parameters from dogs undergoing a graded fitness test with increasing velocity until mild fatigue was achieved.

**Materials and methods:** Thirteen adult dogs, weighing 20–40 kg with a body condition score between 4 and 7/9 and of various degrees of fitness were evaluated. A single training period one week before data collection was used to acclimate dogs to a treadmill. Resting heart rate (HR); systolic (SBP), diastolic (DBP) and mean blood pressure (MBP); rectal temperature; and respiratory rate (RR) were obtained in a quiet room while standing. Dogs underwent a graded exercise test until they reached mild fatigue, defined as unwilling to continue exercising beyond encouragement and pulling back until 1.5 kg of leash tension was achieved. Dogs began walking at 2 km/hr for 10 min, followed by a 1 min rest period to immediately obtain data. Dogs were walked for additional 10 min intervals at increasing velocity (increasing by 0.5 km/hr for each period) until mild fatigue, with 1 min rest periods between sessions for data collection. Data were analyzed to determine if parameters were altered prior to reaching mild fatigue.

**Results:** Dogs exercised for a mean of 50 min (±7.91, range 35–60). Mean resting HR was 87 (±13), which remained relatively stable until 15 min before fatigue, when it increased to 96 (±14). Resting RR was 27 (±5) and gradually increased to 163 (±51) 15 min before fatigue. There were no significant changes in SBP, DBP, MBP, or temperature to indicate fatigue.

**Conclusions:** Mild increases in HR and moderate increases in RR may be useful to help design conditioning protocols to safely result in mild conditioning stress without causing fatigue. Possible recommendations from this graduated exercise test suggest an increase in 10 heartbeats per minute, or a fivefold increase in RR for conditioning stress.

